# Transcriptional regulation of receptor-like protein genes by environmental stresses and hormones and their overexpression activities in *Arabidopsis thaliana*

**DOI:** 10.1093/jxb/erw152

**Published:** 2016-04-19

**Authors:** Jinbin Wu, Zhijun Liu, Zhao Zhang, Yanting Lv, Nan Yang, Guohua Zhang, Menyao Wu, Shuo Lv, Lixia Pan, Matthieu H. A. J. Joosten, Guodong Wang

**Affiliations:** ^1^Key Laboratory of Ministry of Education for Medicinal Plant Resource and Natural Pharmaceutical Chemistry, National Engineering Laboratory for Resource Developing of Endangered Chinese Crude Drugs in Northwest of China, College of Life Sciences, Shaanxi Normal University, Xi’an 710062, China; ^2^Beijing Key Laboratory of Development and Quality Control of Ornamental Horticulture and Landscape Architecture, China Agricultural University, Beijing 100193, China; ^3^Laboratory of Phytopathology, Wageningen University, Droevendaalsesteeg 1, 6708 PB Wageningen, the Netherlands

**Keywords:** Arabidopsis, hormone, overexpression, receptor-like protein, stress, transcriptional regulation.

## Abstract

Receptor-like proteins (RLPs) have been implicated in multiple biological processes, including plant development and immunity to microbial infection. Fifty-seven *AtRLP* genes have been identified in Arabidopsis, whereas only a few have been functionally characterized. This is due to the lack of suitable physiological screening conditions and the high degree of functional redundancy among *AtRLP* genes. To overcome the functional redundancy and further understand the role of *AtRLP* genes, we studied the evolution of *AtRLP* genes and compiled a comprehensive profile of the transcriptional regulation of *AtRLP* genes upon exposure to a range of environmental stresses and different hormones. These results indicate that the majority of *AtRLP* genes are differentially expressed under various conditions that were tested, an observation that will help to select certain *AtRLP* genes involved in a specific biological process for further experimental studies to eventually dissect their function. A large number of *AtRLP* genes were found to respond to more than one treatment, suggesting that one single *AtRLP* gene may be involved in multiple physiological processes. In addition, we performed a genome-wide cloning of the *AtRLP* genes, and generated and characterized transgenic Arabidopsis plants overexpressing the individual *AtRLP* genes, presenting new insight into the roles of *AtRLP* genes, as exemplified by *AtRLP3*, *AtRLP11* and *AtRLP28*. Our study provides an overview of biological processes in which *AtRLP* genes may be involved, and presents valuable resources for future investigations into the function of these genes.

## Highlight

Transcriptional analyses revealed that single *AtRLP* genes may be involved in multiple physiological processes. Overexpression studies found *AtRLP3*/*11* to be involved in meristem development and *AtRLP28* to be involved in salt tolerance.

## Introduction

All living organisms exploit cell-surface receptors to perceive extracellular signals that are from self (e.g. endogenous signaling molecules), non-self (e.g. pathogen-derived molecules) or modified-self (e.g. self molecules that are modified by pathogens) (Cook *et al.*, 2015). In plants, most of these receptors contain extracellular leucine-rich repeats (eLRRs) that are thought to mediate protein–protein interactions (Kobe and Kajava, 2001; Matsubayashi, 2003). Receptor-like proteins (RLPs) represent an important class of such cell-surface receptors. Structurally RLPs consist of two eLRR domains interrupted by an island domain, a single-pass transmembrane domain and a short cytoplasmic tail that lacks obvious motifs for intracellular signaling, except for a putative endocytosis motif found in some members (Tör *et al.*, 2009; Wang *et al.*, 2010a).

RLPs have been shown to play important roles in development and disease resistance in several plant species (Kruijt *et al.*, 2005; Tör *et al.*, 2009; Wang *et al.*, 2010a). Two Arabidopsis RLPs, CLAVATA2 (CLV2)/AtRLP10 and TOO MANY MOUTHS (TMM)/AtRLP17, are known to play a role in plant development. While CLV2 is involved in meristem and organ development, TMM regulates stomatal distribution (Jeong *et al.*, 1999; Nadeau and Sack, 2002; Wang *et al.*, 2008; Wang *et al.*, 2010b; Wang *et al.*, 2011). Apart from CLV2 and TMM, most RLPs characterized to date have been found to be involved in disease resistance. These include the Cf proteins, mediating resistance to the fungal pathogen *Cladosporium fulvum* (Rivas and Thomas, 2005; Thomma *et al.*, 2005; Stergiopoulos and de Wit, 2009); LeEIX, mediating recognition of the ethylene-inducing xylanase (EIX) of the biocontrol fungus *Trichoderma viride* (Ron and Avni, 2004); HcrVf-2, conferring resistance to the apple scab fungus *Venturia inaequalis* (Belfanti *et al.*, 2004); LepR3, providing race-specific resistance to the fungal pathogen *Leptosphaeria maculans* (Larkan *et al.*, 2013); and Ve1, mediating resistance towards *Verticillium* vascular fungi expressing the avirulence gene *Ave1* (Fradin *et al.*, 2009; Fradin *et al.*, 2011).

Over the years, an increasing number of Arabidopsis RLPs (AtRLPs) have been assigned functions in pathogen resistance. We reported previously the assembly of a genome-wide collection of T-DNA insertion lines for the 57 *AtRLP* genes in the Arabidopsis genome (Wang *et al.*, 2008). After an extensive screening only a few novel phenotypes were discovered, including the reported phenotypes for *CLV2* and *TMM*. While AtRLP41 was found to mediate abscisic acid (ABA) sensitivity, AtRLP30 and AtRLP18 were found to influence non-host resistance towards *Pseudomonas syringae* pv. *phaseolicola* (Wang *et al.*, 2008). In addition, AtRLP52 is required for basal defense against the powdery mildew pathogen *Erysiphe cichoracearum* (Ramonell *et al.*, 2005). *SNC2*/*AtRLP51* and *AtRLP55* were suggested to be implicated in basal defense against the bacterial pathogen *Pseudomonas syringae* pv. *tomato* DC3000 (Zhang *et al.*, 2010). ReMAX/AtRLP1 was found to provide recognition of eMAX from Xanthomonads (Jehle *et al.*, 2013), while the fungal pattern sensor RBPG1/AtRLP42 confers resistance to fungal endo-polygalacturonases (Zhang *et al.*, 2014). RFO/AtRLP3 has been implicated in resistance to the vascular wilt fungus *Fusarium oxysporum* forma specialis *matthioli* (Shen and Diener, 2013). As a final example, AtRLP23 was recently found to perceive a conserved 20-amino-acid fragment present in most necrosis and ethylene-inducing peptide (NEP) 1-like proteins, thereby mediating immune activation that, similar to what was observed for the Cf proteins, is dependent on SOBIR1 and SERK3/BAK1 (Liebrand *et al.*, 2013; Albert *et al.*, 2015; Postma *et al.*, 2016). However, the biological functions of the majority of the *AtRLP* genes still remain unclear.

**Fig. 1.**
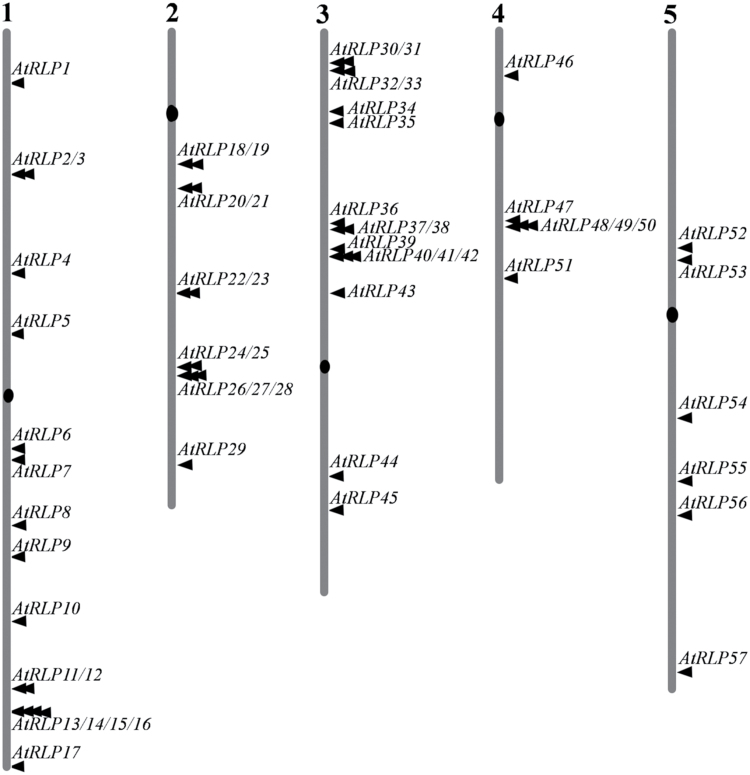
*AtRLP* genes scatter over the different chromosomes of the Arabidopsis genome. The numbers at the top indicate the chromosome number.

The major challenge currently is to understand the biological function of *AtRLP* genes that lack an obvious phenotype in a single mutant background (Wang *et al.*, 2008). One reason is the lack of suitable screening conditions in which the phenotype might only be visible in a condition-specific manner. Interestingly, studies on several *AtRLP* genes have revealed gene expression changes, as well as the emergence of phenotypic alterations, with specific elicitors (Wang *et al.*, 2008; Wang *et al.*, 2010a). Therefore, it may be necessary to test a broad range of physiological conditions, in combination with high-resolution screening for phenotypes. To this end, a comprehensive profile of the transcriptional response of *AtRLP* genes under various conditions, including exposure to biotic and abiotic stress and hormones, will be very helpful. The lack of assignment of biological functions to *AtRLP* genes may also be explained by a strong functional redundancy among the various *AtRLP* genes (Wang *et al.*, 2008). In particular, most of the closely related *AtRLP* genes are located at one locus on the chromosomes (Fig. 1), making it impossible to generate high-order mutant combinations. RNA interference studies that silence multiple *AtRLP* genes simultaneously also failed to uncover new biological functions for several sets of closely related *AtRLP* genes (Ellendorff *et al.*, 2008). As an alternative approach, analysis of the gain-of-function phenotypes has yielded valuable information on the function of *AtRLP* genes, including *TMM*, *Ve1* and *AtRLP23* (Fradin *et al.*, 2009; Fradin *et al.*, 2011; Yan *et al.*, 2014; Albert *et al.*, 2015).

To overcome the functional redundancy and further understand the role of *AtRLP* genes, in this work we studied the evolution of *AtRLP* genes and compiled a comprehensive profile of the transcriptional regulation of *AtRLP* genes upon exposure to environmental stresses and hormones. This will help to select *AtRLP* genes that might be involved in a specific biological process for further experimental studies aimed at dissecting *AtRLP* function. In addition, we performed a genome-wide cloning of *AtRLP* genes, and generated and characterized transgenic Arabidopsis overexpressing individual *AtRLP* genes. The data presented in this study provide valuable resources for future investigations into the biological role of *AtRLP* genes.

## Materials and methods

### Plant materials and growth conditions

The Arabidopsis ecotypes Columbia (Col-0) and Landsberg *erecta* (L*er*) were used as wild-types (WT) for all phenotypic analyses. The *clv2-1* and *rlp10-1* mutants were described previously (Kays and Clark, 1998; Wang *et al.*, 2008). Plants were grown in soil in the greenhouse or on 1/2 MS medium supplemented with 1% sucrose under a 16h light–8h dark regime at 22 °C. For the *in vitro* growth of Arabidopsis plants, seeds were surface-sterilized for 3h by mixing 10mL water, 10mL 99% NaClO and 5mL 99% HCl, and subsequently sown on 1/2 MS solidified with 1% agar. The plates were incubated at 4 °C in the dark for 3 days and transferred to growth chambers.

### Chromosomal locations and analysis of duplication of genes in the *AtRLP* gene family

The chromosomal location of each member of the *AtRLP* family and its location-related *receptor-like kinase* (*RLK*) gene was determined with the Chromosome Map Tool at TAIR (http://www.arabidopsis.org/jsp/ChromosomeMap/tool.jsp). The location of each gene in relation to major chromosomal duplication events in the Arabidopsis genome was determined with tools provided at http://wolfe.gen.tcd.ie/athal/dup and/or defined by Blanc *et al.* (2003). Tandem duplicated genes were identified based on criteria described by Shiu and Bleecker (2003). Briefly, tandem repeats of *AtRLP* genes were defined as genes that are located within 30kb or are separated by five or fewer non-homologous spacer genes.

### Gene expression data analysis

The gene expression data of each experiment presented in this study were obtained from AtGenExpress (http://www.weigelworld.org/resources/microarray/AtGenExpress) (Kilian *et al.*, 2007; Goda *et al.*, 2008), and were normalized using the GC-RMA method (Wu *et al.*, 2004). Fifty-two of the 57 *AtRLP* genes were available in the dataset. The fold-change of expression of *AtRLP* genes under each condition was determined by the expression change relative to the respective controls. Fold-change ratios were subsequently transformed to log_2_ values to indicate the transcript change. We set a two-fold change threshold as a cut-off to identify differentially expressed genes, in which the false discovery rate was found to be around 0.2% (Zhu and Wang, 2000). Overlapping of *AtRLP* gene sets is defined as *AtRLP* genes that display similar responses to the selected conditions.

### *AtRLP* cloning and generation of transgenic plants

The primers to amplify *AtRLP* genes were designed according to the predicted ORF sequences that were retrieved from the TAIR database (https://www.arabidopsis.org). Total RNA was extracted from Arabidopsis seedlings using the EZNA^TM^ Plant RNA Kit (Omega, USA) and reverse-transcribed into cDNA using the RevertAid^TM^ First Strand cDNA Synthesis Kit (MBI, Fermentas, USA). The PCR reaction was conducted using Phusion^TM^ High-Fidelity DNA Polymerase (Finnzymes, Finland). The PCR products were subsequently purified using the Quick DNA Purification Kit (Cwbio, China). The purified PCR products were cloned into *pDONR207* and sequenced. After sequence verification, all entry clones were subsequently recombined into the destination vector pGD625 that contained the CaMV 35S promoter through LR recombination reactions. In the case of *AtRLP32* and *AtRLP46*, CaMV 35S promoters containing destination vectors pFAST-R02 and pB2GW7, respectively, were used.

The resulting constructs were introduced into *Agrobacterium tumefaciens* and transformed into WT plants or the *clv2-1* or *rlp10-1* mutants using the floral dip method (Clough and Bent, 1998). Seeds from transformed plants were selected using corresponding antibiotics until at least three homozygous transgenic lines were obtained for each *AtRLP* gene.

### Stress induction and gene expression analysis by quantitative real-time RT-PCR (qPCR)

The sterilized seeds were sown in 1/2 MS liquid medium containing 1% (w/v) sucrose. After sowing, the medium was incubated at 4 °C in the dark for 3 d and subsequently grown on a roller shaker for 7 d with 16h light–8h dark at 21 °C. For NaCl and mannitol induction, the seedlings were treated with 150mM NaCl and 400mM mannitol, respectively, and sampled at 0, 3, and 6h. The qPCRs were performed in triplicate with SYBR Green PCR Master Mix (Thermo Scientific) using a Bio-Rad IQ5 (Bio-Rad). The *Actin2* gene was used as control to normalize expression levels. For each independent biological replicate, the relative transcript amount was calculated as the mean of three technical replicates. The relative expression levels were normalized to a value of 1 in the respective control samples. All primers used for qPCRs are listed in Supplementary Table S1 at *JXB* online.

### Phenotypic analyses

The carpel number was counted using mature siliques under a dissection microscope. For biological statistics, there was a minimum of 30 plants of which 20 siliques per plant were counted to determine the mean carpel numbers for individual genotypes. For the salt and mannitol tests, seeds were sown on 1/2 MS medium supplemented with different concentrations of salt and mannitol as indicated. The germination rate was analysed in triplicate for each line (around 60 seeds each).

## Results and discussion

### Duplication of *AtRLP* genes in the Arabidopsis genome

We found that *AtRLP* genes are distributed over all five chromosomes with many clusters containing two or more *AtRLP* genes (Fig. 1), suggesting a major role of tandem duplications in the enlargement of the *AtRLP* gene family. We therefore investigated the evolutionary relationship and duplication events of *AtRLP* family members. To this end, we determined the chromosomal locations and the duplicated chromosome segments in which *AtRLP* genes are found (Fig. 1 and Table 1). A total of 35 *AtRLP* genes were found to be present in tandem repeats, representing about 60% of all *AtRLP* genes (Table 1). Out of 57 *AtRLP* genes, 27 were found in the hypothesized duplicated regions, whereas 30 were located outside these regions (Table 1). Within the duplicated regions, 11 *AtRLP* genes were found to have duplicated pair(s), whereas the remaining 16 *AtRLP* genes were found to have no corresponding duplicated pair although their locations were surrounded by duplicated segments (Table 1). Specifically, four pairs of *AtRLP* genes (*AtRLP23* and *AtRLP42*; *AtRLP33* and *AtRLP53*; *AtRLP44* and *AtRLP57*; and *AtRLP51* and *AtRLP55*) constitute pairs of duplicated genes in segmental duplicated blocks of chromosomes. In addition, three *AtRLP* genes, *AtRLP1*, *AtRLP4* and *AtRLP17/TMM*, were found to be duplicated counterparts of the eLRR-containing genes At1g74360 (*eLRR-receptor-like kinase* (*RLK*) gene), At2g14440 (*eLRR-RLK* gene) and At3g126102 (containing an eLRR domain), respectively. No traceable duplication event was found for three genes, namely *AtRLP7*, *AtRLP36*, and *AtRLP52*. Altogether, these results suggest that a major role of the tandem duplications, in conjunction with segmental duplications, has been to contribute to enlargement of the *AtRLP* gene family in Arabidopsis.

**Table 1.** Duplication of AtRLP genes in the Arabidopsis genome

	Outside the duplication region	Within the duplication region	
		With duplicated genes	Without duplicated genes
Singular	3	8	11
Tandem repeats	27	3	5
Total	30	11	16

In a previous study, Shiu and Bleecker (2003) found that some of the *AtRLP* genes locate close to an *RLK*. We identified nine *AtRLP* genes that were located in relatively close proximity (10 predicted genes) to an *RLK* gene (see Supplementary Table S2 at *JXB* online), thus resulting in *RLP*–*RLK* combinations. To determine a possible functional significance of such associations, the expression patterns of these genes were compared using ATTED-II (Obayashi *et al.*, 2014). Unfortunately, co-expression could not be confirmed for any of the *AtRLP*–*RLK* combinations, suggesting that these *RLP*–*RLK* combinations do not have a biological relevance. Indeed, for instance, sequence comparison revealed that *AtRLP52* and At5g25930, encoding an RLK, show a high degree of similarity in their extracellular domains (see Supplementary Fig. S1 at *JXB* online), while they do not exhibit an overlap in expression patterns. It is also possible that several of these *AtRLP* genes that are located in *RLK* gene clusters may have arisen through unequal crossovers (Shiu and Bleecker, 2003), as might be the case for *AtRLP52* and At5g25930.

### *AtRLP* genes display comprehensive and distinct transcriptional regulation upon exposure to external stimuli and hormones

It was suggested that the majority of *AtRLP* genes are involved in plant defense, as has been shown by phylogenomic analysis (Fritz-Laylin *et al.*, 2005). The data suggest potential transcriptional regulation of *AtRLP* genes upon exposure to environmental stimuli. Therefore, we started to investigate the transcriptional regulation of the entire *AtRLP* gene family by external stimuli and hormones. The availability of microarray datasets allowed us to identify specifically regulated *AtRLP* genes. To deepen our understanding of the transcriptional regulation of the *AtRLP* genes, we explored and visualized the expression of the genes under various growth conditions by using AtGenExpress (Kilian *et al.*, 2007). *AtRLP27*, *AtRLP38*, *AtRLP50*, *AtRLP51* and *AtRLP53* are not present in the AtGenExpress, which resulted in a total of 52 *AtRLP* genes that were analysed in our study. *AtRLP8* was found not to exhibit a response to any treatment tested (Supplementary Table S3). An overview of the differentially expressed *AtRLP* genes for all conditions tested can be found in Supplementary Tables S4–S7 at *JXB* online. As the environmental stimuli tested were by no means comprehensive, it is possible that *AtRLP8* expression is responsive to other environmental factors. Only *AtRLP29* was differentially expressed upon various light treatments (Supplementary Table S7), suggesting that the expression of most *AtRLP* genes is not perturbed by light. To confirm the validity of the microarray data, we selected *AtRLP23*, *AtRLP28*, *AtRLP30*, *AtRLP33* and *AtRLP37*, based on our study interests, to examine their expression in response to NaCl and mannitol at different time points. By qPCR, we confirmed that out of 20 samples tested, 14 showed similar expression patterns to the microarray data (Supplementary Fig. S2 at *JXB* online), which represents most of the genes and the two treatments.

**Fig. 2.**
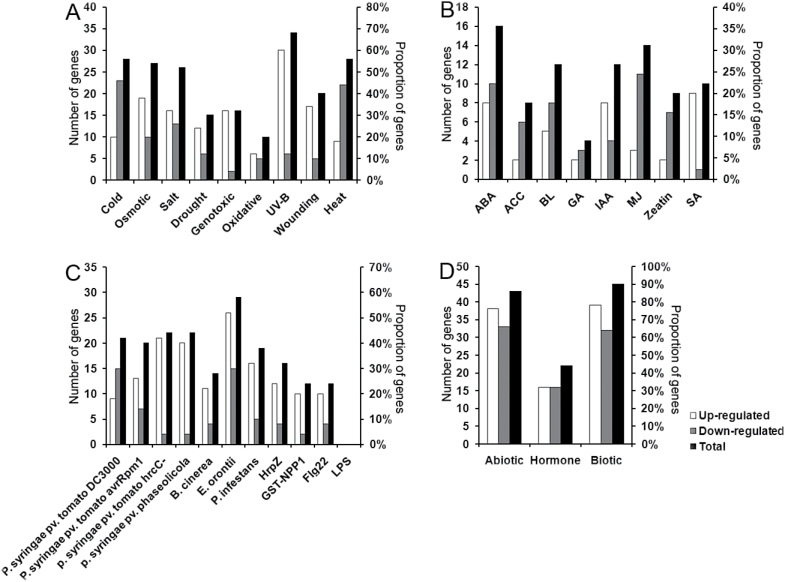
Overview of the amounts of differentially expressed *AtRLP* genes per treatment. The number and proportion of *AtRLP* genes that are differentially expressed per treatment are indicated. An *AtRLP* with a two-fold change or greater is considered as a differentially expressed *AtRLP* gene. (A) Number and proportion of differentially expressed *AtRLP* genes which are regulated by abiotic treatments. (B) Number and proportion of differentially expressed *AtRLP* genes that are regulated by hormonal treatments. (C) Number and proportion of differentially expressed *AtRLP* genes that are regulated by biotic treatments. (D) Number and proportion of differentially expressed *AtRLP* genes in three major classes of treatments, abiotic stress, hormones, and biotic stress. The total number represents the sum of number of up-regulated and down-regulated genes. As a result of dynamic responses, the total number in some cases was smaller than the sum of up-regulated and down-regulated genes.

A total of 51 *AtRLP* genes exhibited differential expression, with a two-fold change or more, under at least one of the tested conditions (Fig. 2, Table 2 and Supplementary Tables S4–S7). A list of *AtRLP* genes showing the highest differential expression per treatment is presented in Table 2. We found that a number of up-regulated *AtRLP* genes were significantly over-represented for multiple stress conditions and hormones tested (Fig. 2, Table 2 and Supplementary Tables S4–S7). In particular, the up-regulated *AtRLP* genes were significantly enriched among seven of the nine abiotic stress conditions and nine of the 11 biotic stress conditions analysed (Fig. 2, Table 2 and Supplementary Tables S4–S7). Amongst all conditions tested, biotic stresses perturbed the expression of the largest proportion of *AtRLP* genes (87%), compared with the abiotic stresses (80%) and hormone treatments (40%) (Fig. 2). In more detail, the largest proportion of differentially expressed *AtRLP* genes was found for the treatments of UV-B, cold, heat, osmotic stress, salt stress, ABA, bacterial pathogens and bacterial pattern HrpZ (Fig. 2 and Supplementary Tables S4–S7).

**Table 2.** The most transcriptionally responsive AtRLP genes per treatment

Treatment	Gene	Accession no.	Log_2_	Treatment	Gene	Accession no.	Log_2_
Cold	*AtRLP33*	At3g05660	7.3	Heat	*AtRLP37*	At3g23110	–3.0
	*AtRLP37*	At3g23110	–3.8		*AtRLP9*	At1g58190	–2.2
	*AtRLP32*	At3g05650	2.8		*AtRLP6*	At1g45616	2.1
	*AtRLP49*	At4g13900	2.6		*AtRLP45*	At3g53240	–2.1
	*AtRLP18*	At2g15040	–2.4		*AtRLP54*	At5g40170	–2.0
Osmotic	*AtRLP33*	At3g05660	5.4	*P. syringae*	*AtRLP22*	At2g32660	5.1
	*AtRLP23*	At2g32680	5.1		*AtRLP21*	At2g25470	4.4
	*AtRLP49*	At4g13900	3.7		*AtRLP20*	At2g25440	4.3
	*AtRLP46*	At4g04220	3.5		*AtRLP40*	At3g24954	4.3
	*AtRLP28*	At2g33080	3.5		*AtRLP26*	At2g33050	–4.1
Salt	*AtRLP33*	At3g05660	4.3	*P. infestans*	*AtRLP19*	At2g15080	2.8
	*AtRLP7*	At1g47890	3.2		*AtRLP30*	At3g05360	2.6
	*AtRLP49*	At4g13900	3.2		*AtRLP49*	At4g13900	2.4
	*AtRLP37*	At3g23110	3.0		*AtRLP18*	At2g15040	2.3
	*AtRLP36*	At3g23010	2.6		*AtRLP46*	At4g04220	2.2
	*AtRLP39*	At3g24900	3.9		*AtRLP18*	At2g15040	–4.6
Drought	*AtRLP37*	At3g23110	3.9	*B. cinerea*	*AtRLP30*	At3g05360	3.1
	*AtRLP18*	At2g15040	–2.2		*AtRLP49*	At4g13900	2.4
	*AtRLP32*	At3g05650	2.1		*AtRLP25*	At2g33030	2.1
	*AtRLP33*	At3g05660	1.9		*AtRLP20*	At2g25440	1.8
Genotoxic	*AtRLP49*	At4g13900	3.8	*E. orontii*	*AtRLP41*	At3g25010	3.7
	*AtRLP37*	At3g23110	3.1		*AtRLP18*	At2g15040	3.2
	*AtRLP23*	At2g32680	2.3		*AtRLP23*	At2g32680	3.1
	*AtRLP7*	At1g47890	1.9		*AtRLP35*	At3g11080	2.9
	*AtRLP34*	At3g11010	1.7		*AtRLP30*	At3g05360	2.6
Oxidative	*AtRLP37*	At3g23110	2.9	HrpZ	*AtRLP30*	At3g05360	3.1
	*AtRLP49*	At4g13900	1.9		*AtRLP22*	At2g32660	3.1
	*AtRLP30*	At3g05360	1.8		*AtRLP21*	At2g25470	3.0
	*AtRLP23*	At2g32680	–1.4		*AtRLP12*	At1g71400	2.6
	*AtRLP41*	At3g25010	–1.4		*AtRLP40*	At3g24954	2.1
UV-B	*AtRLP23*	At2g32680	6.0	GST-NPP1	*AtRLP22*	At2g32660	2.8
	*AtRLP30*	At3g05360	5.5		*AtRLP12*	At1g71400	2.4
	*AtRLP46*	At4g04220	4.8		*AtRLP30*	At3g05360	2.3
	*AtRLP37*	At3g23110	4.8		*AtRLP7*	At1g47890	1.9
	*AtRLP49*	At4g13900	4.7		*AtRLP23*	At2g32680	1.8
Wounding	*AtRLP33*	At3g05660	4.6	Flg22	*AtRLP24*	At2g33020	3.8
	*AtRLP40*	At3g24954	4.1		*AtRLP22*	At2g32660	3.5
	*AtRLP6*	At1g45616	3.5		*AtRLP21*	At2g25470	3.2
	*AtRLP32*	At3g05650	2.3		*AtRLP40*	At3g24954	2.6
	*AtRLP37*	At3g23110	2.1		*AtRLP30*	At3g05360	2.4
ABA	*AtRLP32*	At3g05650	4.0	IAA	*AtRLP23*	At2g32680	2.3
	*AtRLP10*	At1g65380	–3.0		*AtRLP17*	At1g80080	2.0
	*AtRLP12*	At1g71400	–3.0		*AtRLP46*	At4g04220	1.8
	*AtRLP33*	At3g05660	2.7		*AtRLP1*	At1g07390	1.7
	*AtRLP46*	At4g04220	–2.2		*AtRLP21*	At2g25470	1.7
ACC	*AtRLP33*	At3g05660	–2.2	MJ	*AtRLP33*	At3g05660	–2.9
	*AtRLP25*	At2g33030	1.7		*AtRLP1*	At1g07390	–2.5
	*AtRLP1*	At1g07390	1.4		*AtRLP46*	At4g04220	–2.3
	*AtRLP17*	At1g80080	–1.4		*AtRLP22*	At2g32660	2.1
	*AtRLP7*	At1g47890	–1.3		*AtRLP12*	At1g71400	–1.7
BL	*AtRLP23*	At2g32680	2.6	Zeatin	*AtRLP33*	At3g05660	1.9
	*AtRLP33*	At3g05660	–2.6		*AtRLP23*	At2g32680	1.7
	*AtRLP17*	At1g80080	2.4		*AtRLP17*	At1g80080	1.2
	*AtRLP37*	At3g23110	2.1		*AtRLP54*	At5g40170	–1.2
	*AtRLP41*	At3g25010	2.0		*AtRLP46*	At4g04220	–1.1
GA	*AtRLP33*	At3g05660	–2.7	SA	*AtRLP17*	At1g80080	–4.8
	*AtRLP10*	At1g65380	–1.7		*AtRLP34*	At3g11010	3.9
	*AtRLP7*	At1g47890	–1.3		*AtRLP37*	At3g23110	3.6
	*AtRLP37*	At3g23110	1.3		*AtRLP33*	At3g05660	2.7
					*AtRLP30*	At3g05360	1.9

Interestingly, the down-regulated *AtRLP* genes were also enriched in four abiotic conditions and two biotic conditions (Fig. 2, Table 2 and Supplementary Tables S4–S7). Surprisingly, no *AtRLP* gene was differentially expressed upon treatment with the pathogen elicitor lipopolysaccharide (Fig. 2 and Supplementary Table S5). Nevertheless, a number of *AtRLP* genes were differentially expressed under different hormone treatments (Supplementary Table S6). More than half (53%) of the *AtRLP* genes were not perturbed by hormones, whereas only seven and nine *AtRLP* genes were not responsive to biotic and abiotic stresses, respectively (Fig. 2 and Supplementary Table S3). This is consistent with the hypothesis that the majority of *AtRLP* genes are involved in plant stress signaling, as was suggested by phylogenomic analyses (Fritz-Laylin *et al.*, 2005). Indeed, most of the functionally characterized *AtRLP* genes are involved in pathogen defense, whereas only a few of them, e.g. *AtRLP41*, are involved in the response to hormone treatments (Wang *et al.*, 2008).

It was found that with respect to abiotic stresses the most notable perturbation was observed for many *AtRLP* genes in the aerial parts of Arabidopsis seedlings. However, this strong induction was not seen in roots (see Supplementary Table S4 at *JXB* online). These data indicate that the response of *AtRLP* genes to abiotic stresses possibly is tissue or stage specific. In addition, we found that *AtRLP* genes, as a whole, tend to be up-regulated by avirulent *P. syringae*, especially in the case of treatments with the avirulent strains *P. syringae* pv. *tomato* DC3000 HrcC, *P. syringae* pv. *tomato* DC3000 avrRpm1 and *P. syringae* pv. *phaseolicola* (Fig. 2 and Supplementary Table S5). However, many *AtRLP* genes were significantly down-regulated by *P. syringae* pv. *tomato* DC3000, a virulent bacterial strain capable of infecting Arabidopsis (Fig. 2 and Supplementary Table S5). These observations suggest that *AtRLP* genes are involved in basal defense networks that are suppressed by DC3000. Notably, several *AtRLP* genes, including *AtRLP30*, were significantly induced by flg22, a peptide corresponding to the most conserved domain of bacterial flagellin (Supplementary Table S5). It would be of great interest to examine whether those AtRLPs are also involved in mediating flagellin signaling.

Generally, our results indicate that a large number of *AtRLP* genes are responsive to abiotic and biotic stresses, as well as to hormones. In addition, our study provides an overview of the biological processes in which *AtRLP* genes may be involved. Given the fact that phenotypic changes often are observed under suitable physiological conditions, the specific treatments and corresponding *AtRLP* gene expression profiles identified here serve as a resource for targeted screening of individual *AtRLP* genetic mutants.

### Individual *AtRLP* genes are transcriptionally regulated by multiple external stimuli and hormones

It has been suggested previously that some *AtRLP* genes might be responsive to several factors and thereby participate in different signaling pathways (Wang *et al.*, 2008; Fradin *et al.*, 2011). To elucidate this phenomenon, we firstly counted the number of treatments in which individual *AtRLP* genes were differentially expressed across all the treatments tested (see Supplementary Fig. S3 at *JXB* online). Of the 52 *AtRLP* genes, only *AtRLP8* displayed no response to any of the treatments tested (Supplementary Table S3), and six *AtRLP* genes displayed an increase or decrease in expression under only one condition (see Supplementary Fig. S3 and Supplementary Tables S4–S7). The remaining *AtRLP* genes exhibited an increased or decreased expression upon more than one treatment (Supplementary Fig. S3 and Supplementary Tables S4–S7), suggesting that a single *AtRLP* gene may be involved in several physiological processes or in a common process initiated by more than one condition. Our observation that a large number (45 out of 52) of *AtRLP* genes respond to more than one treatment also supports the existence of extensive cross-talk and signal integration among different signaling pathways.

**Fig. 3.**
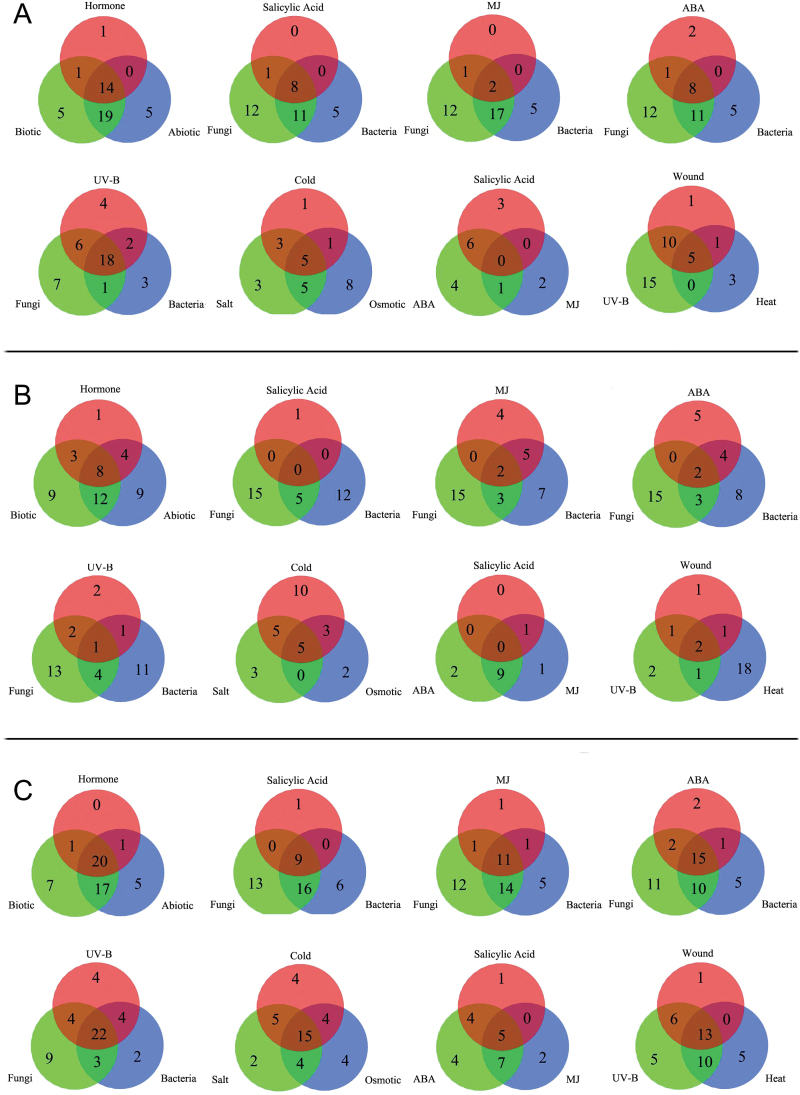
The number of overlapping *AtRLP* genes showing differential regulation in response to different treatments. (A) The number of up-regulated overlapping *AtRLP* genes in three selective treatment sets. (B) The number of down-regulated overlapping *AtRLP* genes in three selective treatment sets as shown in (A). (C) The number of differentially expressed *AtRLP* genes in three selective treatment sets as shown in (A) and (B). Differentially expressed *AtRLP* genes represent the sum of up-regulated and down-regulated genes. As a result of dynamic responses, the number of differentially expressed *AtRLP* genes in some cases was smaller than the sum of up-regulated and down-regulated genes.

Next, we compared sets of three treatments that are known to induce physiological responses to identify overlapping *AtRLP* genes that display multiple responses (Fig. 3). In general, we found a large number of the same *AtRLP* genes had increased expression, while only a few overlapping *AtRLP* genes showed decreased expression upon any of the three treatments we examined (Fig. 3). Thus, no clear stimulus-specific *AtRLP* gene expression patterns could be deduced. Treatment with UV-B, fungi and bacteria showed the largest number of overlapping *AtRLP* genes with increased expression, namely 18, which was followed by 14 shared *AtRLP* genes upon exposure to hormones, biotic and abiotic stresses (Fig. 3). Osmotic stress, salt stress, and cold are intricately linked in various physiological processes (Xiong and Zhu, 2002; Zhu, 2002). With these three treatments there were five *AtRLP* genes with increased response and five *AtRLP* genes with decreased expression (Fig. 3). Eight *AtRLP* genes showed increased expression, while no *AtRLP* showed decreased expression upon exposure to SA, fungi and bacteria (Fig. 3). Among the abiotic stress-related treatments, wounding, UV-B and heat treatment shared five respondents with increased expression and two with decreased expression (Fig. 3). ABA, SA, and methyl jasmonate (MJ) are well known to have cooperative effects in plant development and plant defense (Cutler *et al.*, 2010). Surprisingly, we found that exposure to ABA, SA, and MJ revealed no overlapping genes with either increased or decreased expression (Fig. 3). Similarly, there was very little overlap with either increased or decreased expression found for MJ, fungi, and bacteria (Fig. 3). This may indicate that some of the *AtRLP* genes are conditionally responsive or play a role outside of the cross-talk networks. Nevertheless, the various conditions that we analysed are not exhaustive and more overlap might be found upon additional treatments.

Around ten of the 57 *AtRLP* genes have already been implicated in various physiological programs, as has been discussed (Tör *et al.*, 2009; Wang *et al.*, 2010a). Among them, we found that the expression of *ReMAX*/*AtRLP1*, *RFO*/*AtRLP3*, *AtRLP18*, *AtRLP23*, *AtRLP30* and *RBPG1*/*AtRLP42*, but not *AtRLP55*, is perturbed by a broad set of external stimuli and hormones (Fig. 2 and Supplementary Tables S4–S7). Surprisingly, two developmental *AtRLP* genes, *CLV2* and *TMM*, are differentially expressed upon several external stimuli (Supplementary Tables S4–S7), suggesting dual functions of the encoded proteins in plant development and in the response to stress. For example, *CLV2* is repressed by virulent *P. syringae* pv. *tomato* DC3000 and *Phytophthora infestans*, while *TMM* is repressed by osmotic stress (Supplementary Tables S4–S5). However, *AtPDO2*/*AtRLP4*, another putative developmentally related *AtRLP* gene identified through phylogenomic analysis (Fritz-Laylin *et al.*, 2005), exhibited no alteration in expression under most external conditions tested (Supplementary Tables S4–S7).

In conclusion, our observations reveal that a large number of *AtRLP* genes display differential expression upon more than one treatment, indicating that a single *AtRLP* gene may be involved in several physiological processes. We found that 14 *AtRLP* genes with increased expression and eight *AtRLP* genes with decreased expression, a sum of 22 *AtRLP* genes showing differential expression, are shared among the three major classes of treatments, abiotic stress, biotic stress, and hormones (Fig. 3). The results thus reveal a large number of overlapping *AtRLP* genes responding to the examined treatments. Notably, our analysis also highlights that several known *AtRLP* genes exhibit distinct responses to specific treatments, which has not been described previously.

### Cloning of *AtRLP* genes and sequence analysis of the isolated *AtRLP* genes

The cDNA fragments containing the respective coding sequences of individual *AtRLP* genes were obtained by RT-PCR (Supplementary Tables S8 and S9 at *JXB* online). *AtRLP18* and *AtRLP49* were not amplified, as they were annotated as pseudogenes in the TAIR10 release (see Supplementary Table S9). PCR products were obtained for 51 of the remaining 54 predicted *AtRLP* genes, while *AtRLP1*, *AtRLP8*, *AtRLP15*, and *AtRLP21* failed to amplify by RT-PCR and were excluded from our study (Supplementary Table S9). Purified PCR products were introduced into pDONR207 to produce the entry clones. Plasmid DNA from entry clones was subsequently recombined into the CaMV 35S promoter containing binary vector pGD625, pFAST-R02 or pB2GW7 to generate over-expression constructs.

**Fig. 4.**
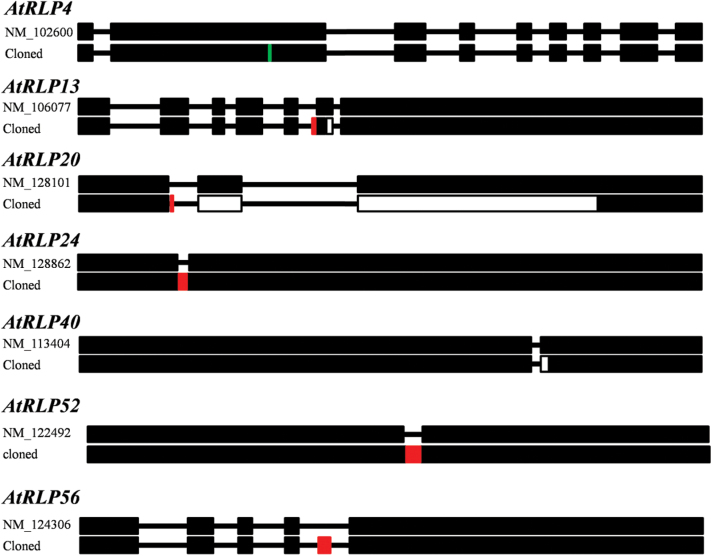
Schematic comparisons of cloned *AtRLP* sequences and predicted mRNA sequences derived from TAIR. Black boxes indicate exons, and lines between exons represent introns. Red boxes represent new exon sequences in the cloned *AtRLP* gene, and open boxes show the missed exon sequences in the cloned *AtRLP* gene. The vertical blue line indicates a single base substitution.

A total of 51 cDNA sequences were successfully cloned into pDONR207 and sequenced (Supplementary Table S9). Among them, 44 out of the 51 isolated clones carrying cDNA sequences were identical to those predicted in TAIR, whereas the other seven isolated sequences differed from their corresponding predictions in TAIR (Fig. 4; Supplementary Table S9). The isolated sequence of *AtRLP4* showed single base substitution, which caused a non-synonymous mutation (Fig. 4 and Supplementary Fig. S4 at *JXB* online). The isolated sequences of *AtRLP13*, *AtRLP20*, and *AtRLP40* showed different intron–exon boundaries as compared with the predicted sequences, resulting in different gene products (Fig. 4 and Supplementary Fig. S4). The predicted introns of *AtRLP24* and *AtRLP52* were eliminated in their isolated sequences and have integrated as a part of the exon, which results in the presence of one single exon instead of two exons (Fig. 4 and Supplementary Fig. S4). An unpredicted exon was found in the isolated sequence of *AtRLP56* (Fig. 4 and Supplementary Fig. S4). These observations indicate that the annotations are probably not correct. It is also possible that the isolated and the predicted sequences are both present *in planta*, which may suggest that some *AtRLP* genes have undergone alternative splicing, probably in different tissues and organs, or upon applying different stimuli.

In this study, a total of 51 *AtRLP* cDNAs containing complete coding sequences were generated, and these will provide useful tools for further functional analyses of this important gene family. For instance, the entry clones can be recombined into any Gateway-compatible destination vector and introduced into Arabidopsis to dissect the resulting phenotypes, which will indicate the possible functions of target genes. It was revealed previously that some conserved residues and motifs of RLPs are of functional significance (Fritz-Laylin *et al.*, 2005; Wang *et al.*, 2008; Wang *et al.*, 2010b). Therefore, the cloned genes could also be mutated in these conserved residues and/or motifs by site-directed mutagenesis to create mutant variants. In line with this hypothesis, it has been shown that transgenic plants expressing a mutation in the conserved GxxxG motif, which is known to aid in protein–protein interactions, that is located on the transmembrane domain of *SNC2*/*AtRLP51* exhibit constitutively activated defense responses (Zhang *et al.*, 2010). In summary, the resources generated in this study will provide useful tools for future functional examination of *AtRLP* genes.

### Generation of transgenic Arabidopsis plants overexpressing *AtRLP* genes

To facilitate functional analysis, we generated transgenic plants overexpressing the individual *AtRLP* genes. To this end, the resulting CaMV 35S-driven expression constructs were transformed into wild-type (Col-0 and/or L*er*) plants and/or the *clv2* mutant (see Supplementary Table S10 at *JXB* online). For each construct, at least 20 independent transgenic lines were initially analysed in the T2 generation, and then at least three independent homozygous lines (T3 or T4 plants) were obtained for each *AtRLP* gene (Supplementary Table S10). Altogether, we generated a collection of 167 homozygous overexpression (*AtRLP-OX*) lines for 51 *AtRLP* genes. This collection of transgenic plants could be used for the analysis of developmental aspects and studies on RLP function in pathogen defense and stress conditions, thus providing a valuable resource for future investigations into the biological role of AtRLPs.

### Overexpression of *AtRLP3* and *AtRLP11* rescues the *clv2-1* mutant

A phenotypical analysis of 4- to 6-week-old homozygous *AtRLP-OX* lines with respect to their growth and development under normal growth conditions did not reveal any abnormalities. Therefore, additional tests need to be performed on these *AtRLP-OX* lines to study the phenotype of various organs at multiple growth and developmental stages.

**Fig. 5.**
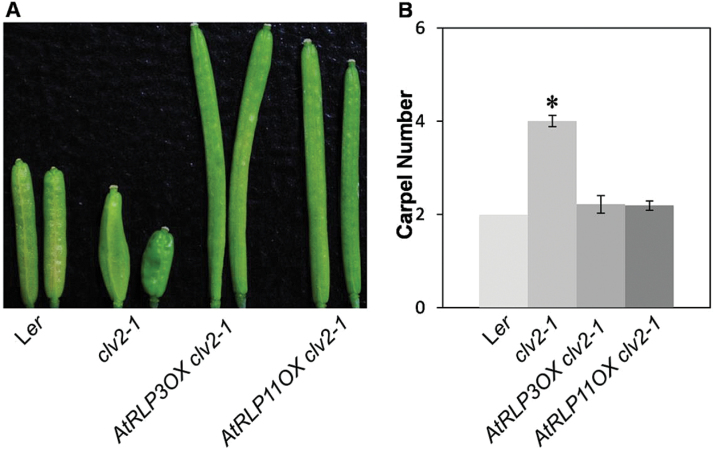
*AtRLP3-OX* and *AtRLP11-OX* rescue the *clv2-1* mutant. (A) Representative siliques of L*er*, *clv2-1*, *AtRLP3-OX* in *clv2-1* and *AtRLP11-OX* in *clv2-1* plants. (B) The mean number of carpels for multiple independent transgenic lines of *AtRLP3-OX* and *AtRLP11-OX* that were transformed into *clv2-1* relative to the wild-type L*er* and the *clv2-1* mutant. For each genotype, a minimum of 30 transgenic plants with 20 siliques per plant were analysed. An asterisk indicates a significant difference (*P*<0.01) compared with the wild-types.

We reported previously that two AtRLPs, AtRLP2 and AtRLP12, which share high sequence similarity to CLV2, are able to rescue the *clv2* mutant phenotype when expressed under the control of the *CLV2* promoter, suggesting that the specialization among CLV2, AtRLP2 and AtRLP12 is largely ascribed to differences in their expression patterns (Wang *et al.*, 2010b). Intriguingly, *AtRLP3* and *AtRLP11* are duplicated genes of *AtRLP2* and *AtRLP12*, respectively, which may suggest a similar function for these paralogues. To test this hypothesis and to investigate the biological role of *AtRLP3* and *AtRLP11*, we analysed the transgenic plants overexpressing *AtRLP3* and *AtRLP11* in either the wild-type plants or the *clv2* mutant (Supplementary Table S10). Wild-type plants developed an invariant two carpels per flower, while *clv2-1* mutants produced multiple carpels per flower (Kayes and Clark, 1998; Wang *et al.*, 2008). Interestingly, *AtRLP3-OX* and *AtRLP11-OX* transformed into the *clv2-1* mutant completely complemented its phenotype, showing a mean carpel number that is comparable to the wild-type plants (Fig. 5), which is similar to what has been shown for *AtRLP2* and *AtRLP12* (Wang *et al.*, 2010b). The overall growth and appearance of *AtRLP3-OX* and *AtRLP11-OX* in the wild-type were indistinguishable from wild-type plants grown under normal growth conditions. Additionally, the *atrlp3-1* and *atrlp11-1* mutants displayed no meristem defects (Wang *et al.*, 2008), despite our observation that *AtRLP3-OX* and *AtRLP11-OX* are able to rescue the phenotype of the *clv2-1* mutant. These results suggest that the functional diversity among these closely related genes is primarily due to divergence of gene expression, rather than of their protein-coding regions. *CLV2* exhibited overlapping expression with *AtRLP2*, *AtRLP3*, *AtRLP11* and *AtRLP12* in some organs, suggesting that *CLV2* may have overlapping functions with these members in those organs.

It has been shown that *RFO2/AtRLP3* confers resistance to the vascular wilt fungus *Fusarium oxysporum*, whereas *AtRLP2* does not (Shen and Diener, 2013). The eLRRs of RFO2/AtRLP3 and AtRLP2 are interchangeable for resistance, while the less conserved membrane-proximal domains of RFO2/AtRLP3 specify resistance (Shen and Diener, 2013). It was thus suggested that *AtRLP2* was a non-functional pseudogene, similar to the case where a loss-of-function polymorphism accounts for the susceptible allele of *Ve1* (Fradin *et al.*, 2009). Conversely, ectopic expression of *AtRLP2* could suppress the *clv2* mutant, suggesting that *AtRLP2* is functional. Combined with our results, this suggests that, unlike AtRLP2, AtRLP3 has a dual function in plant development and immunity (Fig. 5; Shen and Diener, 2013).

### *AtRLP28-OX* lines show enhanced salt stress tolerance in Arabidopsis

In addition to developmental phenotyping, we initiated an assay of the *AtRLP-OX* lines to test the involvement of individual *AtRLP* in the response to salt stress. Our transcriptional analyses have shown that several *AtRLP* genes are responsive to salt (see Supplementary Table S4). However, no evidence is available on their physiological roles in coping with salt stress.

**Fig. 6.**
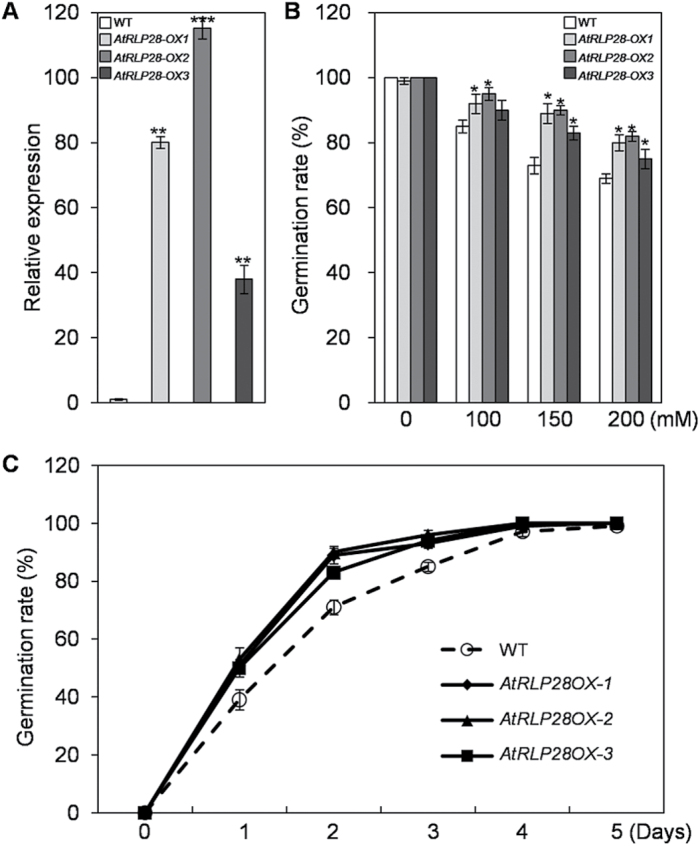
Germination phenotype of the wild-type (WT) and *AtRLP28-OX* lines in response to NaCl treatment. (A) Expression levels of *AtRLP28* in WT and three independent transgenic lines overexpressing *AtRLP28* were determined by qPCR. (B) Germination percentages of WT and three independent *AtRLP28-OX* seeds grown for 2 d on the 1/2 MS medium supplemented with different concentrations of NaCl. Asterisks indicate statistically significant differences compared with WT (* indicates *P*<0.05). (C) Germination percentages of WT and three independent *AtRLP28-OX* seeds grown on 1/2 MS medium containing 150mM NaCl at the indicated times.

To determine whether any *AtRLP* gene plays a role in tolerance to salt stress, we have tested the *AtRLP-OX* lines for their ability to germinate, compared with wild-type seeds, on medium in the presence NaCl. Three independent *AtRLP28-OX* lines exhibited significantly higher germination rates as compared with wild-type seeds (Fig. 6), implying that *AtRLP28* is involved in the tolerance to salt stress. However, the germination rate of *AtRLP28-OX* lines is comparable to that of WT in the presence of mannitol (see Supplementary Fig. S5 at *JXB* online). The elevated expression of *AtRLP28* was confirmed by quantitative RT-PCR for these independent lines (Fig. 6).

To further determine a possible link between *AtRLP28* expression and salt tolerance, the expression of *AtRLP28* was evaluated on exposure to NaCl and mannitol by qPCR. *AtRLP28* transcripts were up-regulated significantly in response to NaCl and mannitol (see Supplementary Fig. S2), which is partially inconsistent with the microarray data. The discrepancy may be due to the difference in the samples collected. Indeed, in a previous study, *AtRLP28* expression was shown to be significantly up-regulated under NaCl treatment (Jung *et al.*, 2008). The data thus confirm our qPCR analyses. In conclusion, these results indicate that high levels of *AtRLP28* expression enhance plant salt tolerance. However, how *AtRLP28* mediates salt stress tolerance requires further biological investigation.

### Conclusions

The Arabidopsis genome contains 57 *AtRLP* genes, the majority of which have yet to be assigned biological roles. In this study, we compiled a detailed expression profile of the transcriptional regulation of *AtRLP* genes upon exposure to a broad range of environmental stresses and hormones. Our results indicate that a large number of *AtRLP* genes are differentially regulated upon various conditions tested, thus providing an overview of the processes in which *AtRLP* genes may be involved. Furthermore, our data revealed that a large number of *AtRLP* genes display differential expression upon more than one treatment, indicating that a single *AtRLP* gene may be involved in multiple physiological processes. The specific processes and the alteration of the expression of the corresponding *AtRLP* genes identified here serve as a tool for targeted screenings of individual *AtRLP* mutants and *AtRLP*-OX lines. In addition, we performed a genome-wide cloning of *AtRLP* genes, and generated and characterized transgenic plants overexpressing individual *AtRLP* genes. As an initial attempt to elucidate the biological role of *AtRLP* genes using these *AtRLP*-*OX* lines, we found that *AtRLP3-OX* and *AtRLP11-OX* are able to rescue the phenotype of the *clv2-1* mutant, which indicates that, similar to their duplicated genes *AtRLP2* and *AtRLP12*, the functional specificity of these genes is determined at the level of their transcriptional regulation. Furthermore, *AtRLP28* was found to mediate salt stress tolerance. Taken as a whole, the comprehensive profile and the generated *AtRLP*-OX lines provide valuable resources for future investigations into the biological role of *AtRLP* genes.

## Supplementary data

Supplementary data are available at JXB online.

Figure S1. The sequence comparison of the extracellular domains of AtRLP52 and At5g25930.

Figure S2. The expression of *AtRLP23*, *AtRLP28*, *AtRLP30*, *AtRLP33* and *AtRLP37* in response to NaCl and mannitol at indicated times.

Figure S3. Number of treatments in which a given *AtRLP* gene is up-regulated, down-regulated and differentially expressed.

Figure S4. Sequence comparisons of cloned *AtRLP* sequences, genomic DNA sequences and predicted mRNA sequences derived from TAIR.

Figure S5. Osmotic effects on the seeds germination of WT and *AtRLP28-OX* lines using mannitol.

Table S1. A list of quantitative real-time PCR primers used in this study.

Table S2. *AtRLP* genes which locate close to an *RLK* gene.

Table S3. *AtRLP* genes displaying no transcriptional responses to the experimental conditions.

Table S4. Gene expression of *AtRLP*s under abiotic stress.

Table S5. Gene expression of *AtRLP*s under biotic stress.

Table S6. Gene expression of *AtRLP*s upon treatment with hormones.

Table S7. Gene expression of *AtRLP*s under different light conditions.

Table S8. A list of primers used in the cloning of *AtRLP* genes.

Table S9. Overview of the cloning results of *AtRLP* genes.

Table S10. Summary of the *AtRLP-OX* transgenic plants.

Supplementary Data
