# Supplementary material for: Transcriptional regulation of receptor-like protein genes by environmental stresses and hormones and their overexpression activities in Arabidopsis thaliana
Source: J Exp Bot. 2016 Apr 19;67(11):3339–51. doi: 10.1093/jxb/erw152 (PMC4892725; doi:10.1093/jxb/erw152)
Supplement: Supplementary Data [file supp_67_11_3339__index.html]

Transcriptional regulation of receptor-like protein genes by environmental stresses and hormones and their overexpression activities in Arabidopsis thaliana — Transcriptional regulation of receptor-like protein genes by environmental stresses and hormones and their overexpression activities in Arabidopsis thaliana — Supplementary Data 

# Transcriptional regulation of receptor-like protein genes by environmental stresses and hormones and their overexpression activities in *Arabidopsis thaliana*

## Supplementary Data

Data files

- Supplementary Data - Supplementary Data
- Supplementary Data - Supplementary Data
- Supplementary Data - Supplementary Data
- Supplementary Data - Supplementary Data
- Supplementary Data - Supplementary Data
- Supplementary Data - Supplementary Data
