# Supplementary material for: Transcriptional regulation of receptor-like protein genes by environmental stresses and hormones and their overexpression activities in Arabidopsis thaliana
Source: J Exp Bot. 2016 Apr 19;67(11):3339–51. doi: 10.1093/jxb/erw152 (PMC4892725; doi:10.1093/jxb/erw152)
Supplement: Supplementary Data [file supp_erw152_ERW152_New_Figures_S1_S5_Tables_S1_S3_Tables_S9_S10.pdf]

<u>Transcriptional</u> Regulation of Receptor-Like Protein Genes by Environmental Stress and Hormones and Their Overexpression Activities in *Arabidopsis thaliana*

Jinbin Wu[1], Zhijun Liu[1], Zhao Zhang, Yanting Lv, Nan Yang, Guohua Zhang, Menyao Wu, Shuo Lv, Lixia Pan, Matthieu H. A. J. Joosten, Guodong Wang[*]

Supplemental files: Figures S1-S5; Tables S1-S3; Tables S9-S10

Figure. S1. The sequence comparison of the extracellular domains of AtRLP52 and At5g25930. Amino acid sequence similarity is indicated by shading (black for 100% conservation, gray for ⩾55% conservation).

```
AtRLP52    MTFLPLLFIFFFLTSIPFPAFSQYNDRSTLLNLKRDLGDPLSLRLWNDTSSPCNWPRITCTAGNVTEINFQNQNFTGTVPTTICNFPNLKSLNLSFNYFAGEFP
At5g25930  MTRLPLPFLFFFLTSIPLSVFSQFNDQSTLLNLKRDLGDPPSLRLWNNTSSPCNWSEITCTAGNVTGINFKNQNFTGTVPTTICDLSNLNFLDLSFNYFAGEFP

AtRLP52    TVLYNCTKLQYLDLSQNLFNGSLPDDINRLAPKLKYLDLAANSFAGDIPKNIGRISKLKVLNLYMSEYDGTFPSEIGDLSELEELQLALNDKFTPVKLPTEFGK
At5g25930  TVLYNCTKLQYLDLSQNLLNGSLPVDIDRLSPELDYLDLAANGFSGDIPKSLGRISKLKVLNLYQSEYDGTFPSEIGDLSELEELRLALNDKFTPAKIPIEFGK

AtRLP52    LKKLKYMWLEEMNLIGEISAVVFENMTDLKHVDLSVNNLTGRIPDVLFGLKNLTELYLFANDLTGEIPKSISAKNLVHLDLSANNLNGSIPESIGNLTNLELLY
At5g25930  LKKLKYMWLEEMNLIGEISPVVFENMTDLEHVDLSVNNLTGRIPDVLFGLKNLTEFYLFANGLTGEIPKSISATNLVFLDLSANNLTGSIPVSIGNLTKLQVLN

AtRLP52    LFVNELTGEIPRAIGKLPELKELKLFTNKLTGEIPAEIGFISKLERFEVSENQLTGKLPENLCHGGKLQSVIVYSNNLTGEIPESLGDCETLSSVLLQNNGFSG
At5g25930  LFNNKLTGEIPPVIGKLPGLKEFKIFNNKLTGEIPAEIGVHSKLERFEVSENQLTGKLPENLCKGGKLQGVVVYSNNLTGEIPESLGDCGTLLTVQLQNNDFSG
```

Figure S2. The expression of *AtRLP23*, *AtRLP28*, *AtRLP30*, *AtRLP33* and *AtRLP37* in response to NaCl and mannitol at the indicated times. Transcript levels were quantified by qRT-PCR using *Actin2* as a control. To validate the qPCR analyses, *RD29A* or *RAB18* was used as a positive control for the NaCl or mannitol treatment, respectively. The time point was indicated on the X-axis. Data shown are means ±SD of three independent experiments. Asterisks indicate statistically significant differences compared with controls (* indicates P< 0.05; ** indicates P<0.01; *** indicates P<0.001).

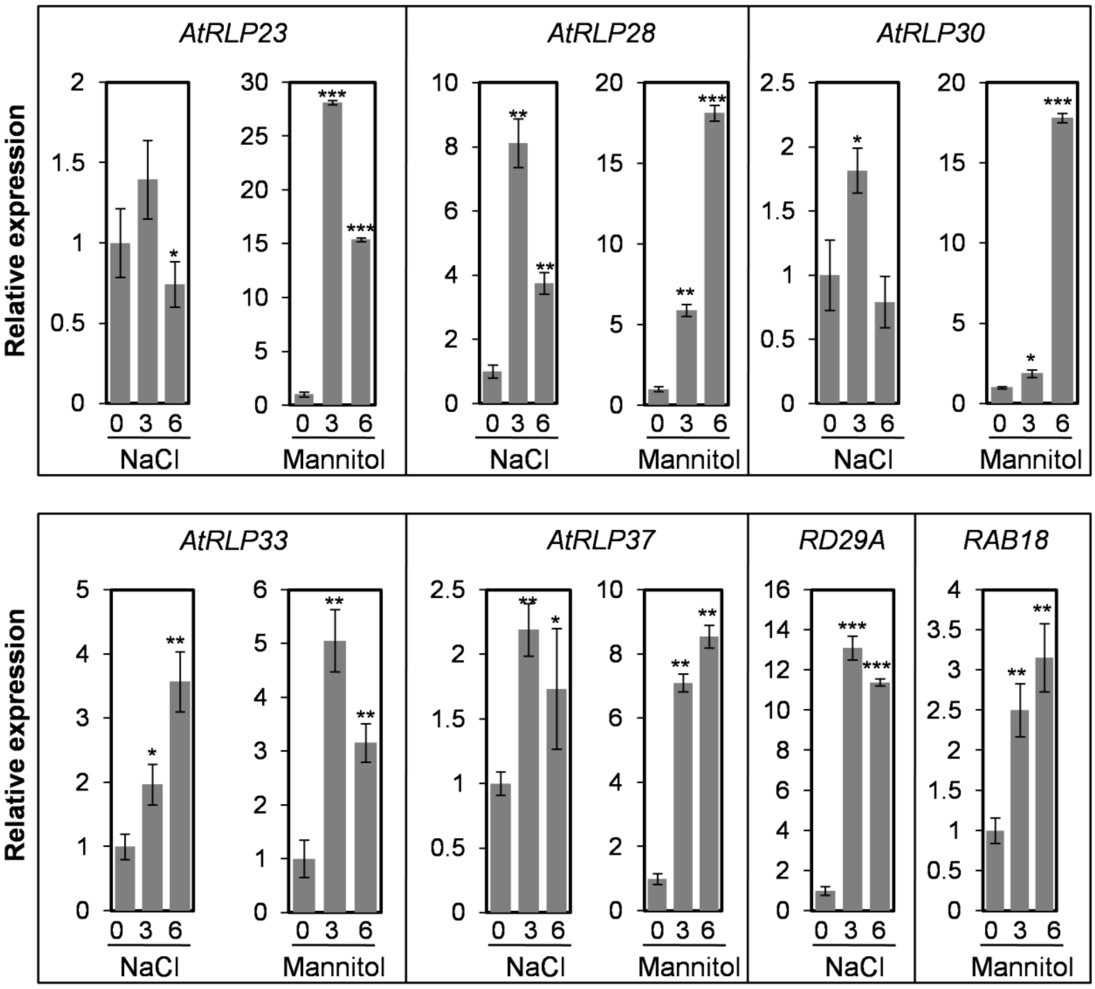

Figure S3. Number of treatments in which a given *AtRLP* gene is up-regulated, down-regulated and differentially expressed.

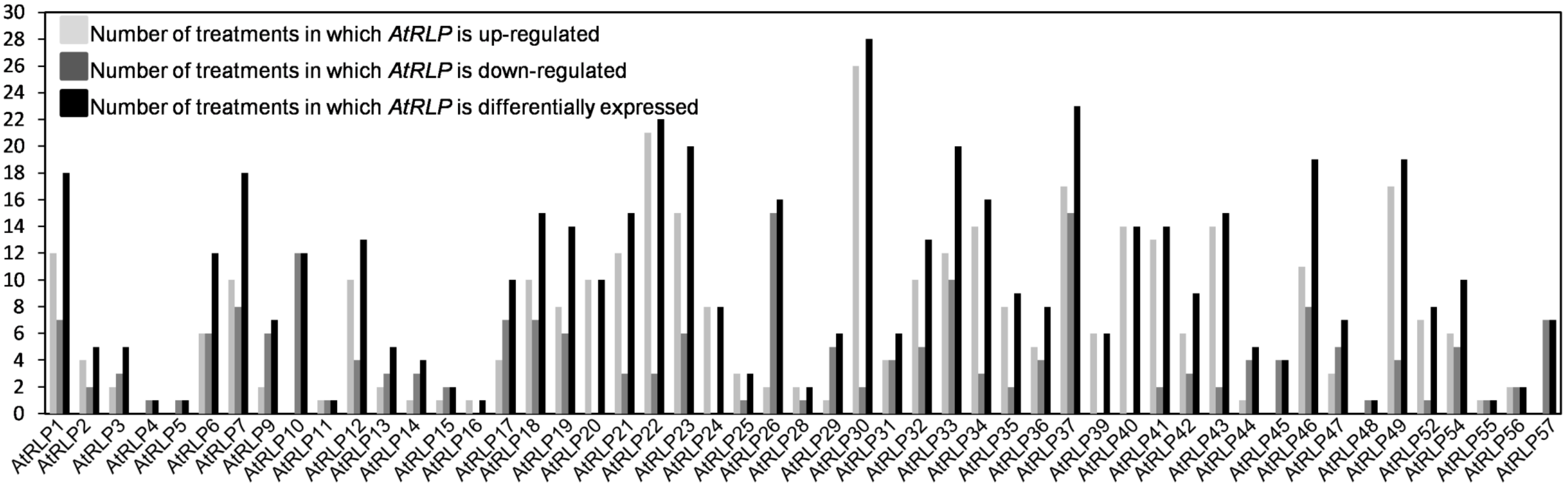

Figure S4. Sequence comparisons of cloned *AtRLP* sequences, genomic DNA sequences and predicted mRNA sequences derived from TAIR. Sequences that are differ with the TAIR sequences are boxed.

AtRLP4

```
                   *        20         *        40         *        60         *        80         *       100         *       120         *       140
Genomic-Seq : ATGATGCTTCGATTTATCCTAGCTTCTCTTCTCCTTTCCTCTTTTAGTCTCTACTCTTCTCTAGCTCGACCAGGTTCGTTTTCTAGATTTAGTTTCTTCTGATTCGAAATCTTTTGACTTGCATTTGACTTTGATTCGCT  140
NM_102600   : ATGATGCTTCGATTTATCCTAGCTTCTCTTCTCCTTTCCTCTTTTAGTCTCTACTCTTCTCTAGCTCGACCAG-------------------------------------------------------------------   73
Cloned      : ATGATGCTTCGATTTATCCTAGCTTCTCTTCTCCTTTCCTCTTTTAGTCTCTACTCTTCTCTAGCTCGACCAG-------------------------------------------------------------------   73

                   *       160         *       180         *       200         *       220         *       240         *       260         *       280
Genomic-Seq : TGATTTGCTTAGCTCCATATGCCTTGAGGATTAGTTGTGGAGCTAGGAAGAATGTTCGAACACCACCAACTTACGCACTTTGGTTCAAGGATATAGCATATACAGGAGGCGTACCTGCCAATGCAACAACGCCTACTTAC  280
NM_102600   : ------------CTCCATATGCCTTGAGGATTAGTTGTGGAGCTAGGAAGAATGTTCGAACACCACCAACTTACGCACTTTGGTTCAAGGATATAGCATATACAGGAGGCGTACCTGCCAATGCAACAACGCCTACTTAC  201
Cloned      : ------------CTCCATATGCCTTGAGGATTAGTTGTGGAGCTAGGAAGAATGTTCGAACACCACCAACTTACGCACTTTGGTTCAAGGATATAGCATATACAGGAGGCGTACCTGCCAATGCAACAACGCCTACTTAC  201

                   *       300         *       320         *       340         *       360         *       380         *       400         *       420
Genomic-Seq : ATAACTCCTCCTTTGAAGACTCTTAGATATTTCCCAATCTCTGAAGGACCTAACAATTGCTACAACATTGTCAGAGTTCCTAAGGGTCATTACTCTGTCAGGATCTTCTTTGGACTTGTTGATCAACCTAGTTTTGATAA  420
NM_102600   : ATAACTCCTCCTTTGAAGACTCTTAGATATTTCCCAATCTCTGAAGGACCTAACAATTGCTACAACATTGTCAGAGTTCCTAAGGGTCATTACTCTGTCAGGATCTTCTTTGGACTTGTTGATCAACCTAGTTTTGATAA  341
Cloned      : ATAACTCCTCCTTTGAAGACTCTTAGATATTTCCCAATCTCTGAAGGACCTAACAATTGCTACAACATTGTCAGAGTTCCTAAGGGTCATTACTCTGTCAGGATCTTCTTTGGACTTGTTGATCAACCTAGTTTTGATAA  341

                   *       440         *       460         *       480         *       500         *       520         *       540         *       560
Genomic-Seq : AGAACCTCTCTTTGACATTTCTATTGAAGGCACTCAGATTTCTTCTCTTAAATCTGGTTGGAGTAGTCAAGATGATCAAGTTTTCGCTGAAGCTCTCATTTTTCTTTTGGGTGGGACTGCTACTATCTGTTTTCATAGTA  560
NM_102600   : AGAACCTCTCTTTGACATTTCTATTGAAGGCACTCAGATTTCTTCTCTTAAATCTGGTTGGAGTAGTCAAGATGATCAAGTTTTCGCTGAAGCTCTCATTTTTCTTTTGGGTGGGACTGCTACTATCTGTTTTCATAGTA  481
Cloned      : AGAACCTCTCTTTGACATTTCTATTGAAGGCACTCAGATTTCTTCTCTTAAATCTGGTTGGAGTAGTCAAGATGATCAAGTTTTCGCTGAAGCTCTCATTTTTCTTTTGGGTGGGACTGCTACTATCTGTTTTCATAGTA  481

                   *       580         *       600         *       620         *       640         *       660         *       680         *       700
Genomic-Seq : CTGGTCATGGCGATCCTGCGATTTTGAGTATTGAGATTCTTCAGGTTGATGATAAAGCTTACAGCTTTGGTGAAGGTTGGGGTCAAGGAGTGATTTTAAGAACTGCCACTAGGTTGACTTGTGGTACTGGGAAGTCTAGG  700
NM_102600   : CTGGTCATGGCGATCCTGCGATTTTGAGTATTGAGATTCTTCAGGTTGATGATAAAGCTTACAGCTTTGGTGAAGGTTGGGGTCAAGGAGTGATTTTAAGAACTGCCACTAGGTTGACTTGTGGTACTGGGAAGTCTAGG  621
Cloned      : CTGGTCATGGCGATCCTGCGATTTTGAGTATTGAGATTCTTCAGGTTGATGATAAAGCTTACAGCTTTGGTGAAGGTTGGGGTCAAGGAGTGATTTTAAGAACTGCCACTAGGTTGACTTGTGGTACTGGGAAGTCTAGG  621

                   *       720         *       740         *       760         *       780         *       800         *       820         *       840
Genomic-Seq : TTTGATGAGGATTATAGAGGGGATCATTGGGGTGGTGATAGGTTTTGGAATCGTATGAGATCTTTTGGTAAGAGTGCTGATTCTCCTAGATCAACTGAGGAAACTATTAAGAAGGCTTCAGTTTCACCAAATTTTTATCC  840
NM_102600   : TTTGATGAGGATTATAGAGGGGATCATTGGGGTGGTGATAGGTTTTGGAATCGTATGAGATCTTTTGGTAAGAGTGCTGATTCTCCTAGATCAACTGAGGAAACTATTAAGAAGGCTTCAGTTTCACCAAATTTTTATCC  761
Cloned      : TTTGATGAGGATTATAGAGGGGATCATTGGGGTGGTGATAGGTTTTGGAATCGTATGAGATCTTTTGGTAAGAGTGCTGATTCTCCTAGATCAACTGAGGAAACTATTAAGAAGGCTTCAGTTTCACCAAATTTTTATCC  761

                   *       860         *       880         *       900         *       920         *       940         *       960         *       980
Genomic-Seq : AGAAGGACTTTATCAGTCTGCGTTGGTTAGTACTGACGACCAACCTGATTTAACGTATAGCTTGGATGTGGAACCCAACAGAAATTACTCTGTATGGTTGCACTTTGCAGAGATTGATAATACTATCACTGCTGAAGGAA  980
NM_102600   : AGAAGGACTTTATCAGTCTGCGTTGGTTAGTACTGACGACCAACCTGATTTAACGTATAGCTTGGATGTGGAACCCAACAGAAATTACTCTGTATGGTTGCACTTTGCAGAGATTGATAATACTATCACTGCTGAAGGAA  901
Cloned      : AGAAGGACTTTATCAGTCTGCGTTGGTTAGTACTGACGACCAACCTGATTTAACGTATAGCTTGGATGTGGAACCCAACAGAAATTACTCTGTATGGTCGCACTTTGCAGAGATTGATAATACTATCACTGCTGAAGGAA  901

                   *      1000         *      1020         *      1040         *      1060         *      1080         *      1100         *      1120
Genomic-Seq : AAAGGGTGTTTGATGTTGTGATAAATGGAGATACTTTCTTTGAAGATGTGGATATCATAAAGATGAGTGGTGGTCGTTATGCAGCACTTGTTCTTAATGCAACAGTGACTGTGAGTGGAAGAACCTTAACTGTCGTTCTA 1120
NM_102600   : AAAGGGTGTTTGATGTTGTGATAAATGGAGATACTTTCTTTGAAGATGTGGATATCATAAAGATGAGTGGTGGTCGTTATGCAGCACTTGTTCTTAATGCAACAGTGACTGTGAGTGGAAGAACCTTAACTGTCGTTCTA 1041
Cloned      : AAAGGGTGTTTGATGTTGTGATAAATGGAGATACTTTCTTTGAAGATGTGGATATCATAAAGATGAGTGGTGGTCGTTATGCAGCACTTGTTCTTAATGCAACAGTGACTGTGAGTGGAAGAACCTTAACTGTCGTTCTA 1041

                   *      1140         *      1160         *      1180         *      1200         *      1220         *      1240         *      1260
Genomic-Seq : CAACCCAAGGCAGGTGGTCATGCTATTATCAATGCCATTGAGGTCTTTGAAATCATAACCGCGGAGTTTAAAACATTACGGGATGAAGGTACACATTCTTTCATGGACACTTTTTAATGTTGTATTTTCACCGCTCTTCG 1260
NM_102600   : CAACCCAAGGCAGGTGGTCATGCTATTATCAATGCCATTGAGGTCTTTGAAATCATAACCGCGGAGTTTAAAACATTACGGGATGAAG---------------------------------------------------- 1129
Cloned      : CAACCCAAGGCAGGTGGTCATGCTATTATCAATGCCATTGAGGTCTTTGAAATCATAACCGCGGAGTTTAAAACATTACGGGATGAAG---------------------------------------------------- 1129

                   *      1280         *      1300         *      1320         *      1340         *      1360         *      1380         *      1400
Genomic-Seq : TTTTCCTGCATAGATTCATATAAAGTTTGGTTACTTGAAAACGAGACATGTGTGCCTCTTGTCGTCAGCTGTCTTTCTACTGTTGTAGAATGGTATTTGGTTTATATGTAATGTGTGATTCAAGTAAATCTTATTCGTAT 1400
NM_102600   : --------------------------------------------------------------------------------------------------------------------------------------------    -
Cloned      : --------------------------------------------------------------------------------------------------------------------------------------------    -

                   *      1420         *      1440         *      1460         *      1480         *      1500         *      1520         *      1540
Genomic-Seq : TTGTGCTAATCTAAGTAGGAATACAGTTCTGATGTTGGAGAAAACTTGTCACTCATGGTTTATTCTTCTAATAGCTAAGCTGGTTGATTGCTTTCTGTATCACTAGATTATTATAATTGTACATAATGTTGCTTGTTGTT 1540
NM_102600   : --------------------------------------------------------------------------------------------------------------------------------------------    -
Cloned      : --------------------------------------------------------------------------------------------------------------------------------------------    -

                   *      1560         *      1580         *      1600         *      1620         *      1640         *      1660         *      1680
Genomic-Seq : TTCTGTTTCAGTCAGTGCGTTACAGAAAATGAAAAAAGCTTTAGGACTTCCTTCTAGGTTTGGATGGAATGGTGATCCATGTGTTCCCCCACAACATCCATGGAGCGGGGCTAATTGTCAGTTGGACAAGAACACCAGCA 1680
NM_102600   : -----------TCAGTGCGTTACAGAAAATGAAAAAAGCTTTAGGACTTCCTTCTAGGTTTGGATGGAATGGTGATCCATGTGTTCCCCCACAACATCCATGGAGCGGGGCTAATTGTCAGTTGGACAAGAACACCAGCA 1258
Cloned      : -----------TCAGTGCGTTACAGAAAATGAAAAAAGCTTTAGGACTTCCTTCTAGGTTTGGATGGAATGGTGATCCATGTGTTCCCCCACAACATCCATGGAGCGGGGCTAATTGTCAGTTGGACAAGAACACCAGCA 1258

                   *      1700         *      1720         *      1740         *      1760         *      1780         *      1800         *      1820
Genomic-Seq : GATGGTTCATTGATGGACTGTATAGCTCGTTTCTCTTCTTTTTGTGTATACTCATAAATAACAGATTCATTAACCCTTTCTTTTGCTTAGGCATCTCATTAGTTAGAGTCTTAGAGTCAAAGGATCTCTCTTTGAGTTTC 1820
NM_102600   : GATGGTTCATTGATGGACT------------------------------------------------------------------------------------------------------------------------- 1277
Cloned      : GATGGTTCATTGATGGACT------------------------------------------------------------------------------------------------------------------------- 1277

                   *      1840         *      1860         *      1880         *      1900         *      1920         *      1940         *      1960
Genomic-Seq : TGGTCTTTTATCAGGTTTTCTAATAAACAAACTTTGTTTTTCGTTGCAGTGATCTTGATAACCAAGGTCTGAAAGGTTTCCTACCAAACGATATATCAAAGCTAAAACATCTTCAGAGCATGTAAGTCAATACATTATGC 1960
NM_102600   : -------------------------------------------------TGATCTTGATAACCAAGGTCTGAAAGGTTTCCTACCAAACGATATATCAAAGCTAAAACATCTTCAGAGCAT------------------- 1349
Cloned      : -------------------------------------------------TGATCTTGATAACCAAGGTCTGAAAGGTTTCCTACCAAACGATATATCAAAGCTAAAACATCTTCAGAGCAT------------------- 1349

                   *      1980         *      2000         *      2020         *      2040         *      2060         *      2080         *      2100
```

```
Genomic-Seq : ACATCCTGCAAATGTTTATCCAAGTGTCTACGAATATTGTATGTAAACGAACAATTTTGTTATCTTCTCCAAGAAAATGACTTGTCTAATGAAGAAATTGTGTCCATTTTTCTTGTGTGTCATTAGTTTTTCTTCCCTCA  2100
NM_102600   : --------------------------------------------------------------------------------------------------------------------------------------------     -
Cloned      : --------------------------------------------------------------------------------------------------------------------------------------------     -

                      *        2120         *        2140         *        2160         *        2180         *        2200         *        2220         *        2240
Genomic-Seq : TTTGTGTAATCCCCCTTACAGTGAAAGCTTCTTGATCCATATCTTGTCCATATTTCAGAAACTTGAGTGAAAACAACATTCGTGGAGGAATACCTGCATCGCTTGGAAGCGTTACGAGCTTGGAAGTATTGTAAGTAATC  2240
NM_102600   : ----------------------------------------------------------AAACTTGAGTGAAAACAACATTCGTGGAGGAATACCTGCATCGCTTGGAAGCGTTACGAGCTTGGAAGTATTG---------  1422
Cloned      : ----------------------------------------------------------AAACTTGAGTGAAAACAACATTCGTGGAGGAATACCTGCATCGCTTGGAAGCGTTACGAGCTTGGAAGTATTG---------  1422

                      *        2260         *        2280         *        2300         *        2320         *        2340         *        2360         *        2380
Genomic-Seq : AGTTTGTTTATTAATTGAGTACTTTATGTGTTAACTTTTCAATTCTGAACCCTGAAACTGAATTTTTATGCATCTTTGCAGGGACCTCTCCTACAATTCCTTCAATGGATCTATCCCTGAAACCTTAGGAGAGTTAACAT  2380
NM_102600   : ----------------------------------------------------------------------------------GACCTCTCCTACAATTCCTTCAATGGATCTATCCCTGAAACCTTAGGAGAGTTAACAT  1480
Cloned      : ----------------------------------------------------------------------------------GACCTCTCCTACAATTCCTTCAATGGATCTATCCCTGAAACCTTAGGAGAGTTAACAT  1480

                      *        2400         *        2420         *        2440         *        2460         *        2480         *        2500         *        2520
Genomic-Seq : CCCTGCGGATTCTGTAAGATTATCAACTTTCTGTTTGTCTCCATGTCAATTTCAGTGGCCAGTGGAGTTCACATGGTCGGCAACATGAAACCTTTTATTGTATTACAGGAATCTCAACGGAAACTCTTTGTCTGGAAAAG  2520
NM_102600   : CCCTGCGGATTCTG-----------------------------------------------------------------------------------------------AATCTCAACGGAAACTCTTTGTCTGGAAAAG  1525
Cloned      : CCCTGCGGATTCTG-----------------------------------------------------------------------------------------------AATCTCAACGGAAACTCTTTGTCTGGAAAAG  1525

                      *        2540         *        2560         *        2580         *        2600         *        2620         *        2640         *        2660
Genomic-Seq : TACCAGCGGCTGTAGGTGGAAGGCTGTTGCACAGAGCAAGCTTCAAGTAAGATGATTGAATCAGAGACAAAGTTTTTAAAGAAATATTTGGACTTGGCATGTGAAGAATGATGTTTGGCAAATAGCACTCACTACCCTGT  2660
NM_102600   : TACCAGCGGCTGTAGGTGGAAGGCTGTTGCACAGAGCAAGCTTCAA----------------------------------------------------------------------------------------------  1571
Cloned      : TACCAGCGGCTGTAGGTGGAAGGCTGTTGCACAGAGCAAGCTTCAA----------------------------------------------------------------------------------------------  1571

                      *        2680         *        2700         *        2720         *        2740         *        2760         *        2780         *        2800
Genomic-Seq : AACTGTTTTCTCTATCTCTGCAGCTTCACAGATAATGCAGGTCTTTGCGGAATCCCGGGGCTACCAGCTTGCGGGCCTCACCTTTCTTCTGGAGCCAAGATTGGCATTGCATTCGGTGTTAGTCTAGCATTCTTATTGAT  2800
NM_102600   : -----------------------CTTCACAGATAATGCAGGTCTTTGCGGAATCCCGGGGCTACCAGCTTGCGGGCCTCACCTTTCTTCTGGAGCCAAGATTGGCATTGCATTCGGTGTTAGTCTAGCATTCTTATTGAT  1688
Cloned      : -----------------------CTTCACAGATAATGCAGGTCTTTGCGGAATCCCGGGGCTACCAGCTTGCGGGCCTCACCTTTCTTCTGGAGCCAAGATTGGCATTGCATTCGGTGTTAGTCTAGCATTCTTATTGAT  1688

                      *        2820         *        2840         *        2860         *        2880         *        2900         *        2920         *        2940
Genomic-Seq : CGTTGCATGTGCAATGATATGGTGGAAACGACGACAGAATATTCTTCGGGCACAGCAGATTGCAGGTAAACATTCATTTCCGTCACTAGGAAGTTGTATTTAGTCTTTTAAAACAAACCTTTATGAGCCTGCACTGTGAA  2940
NM_102600   : CGTTGCATGTGCAATGATATGGTGGAAACGACGACAGAATATTCTTCGGGCACAGCAGATTGCAG---------------------------------------------------------------------------  1753
Cloned      : CGTTGCATGTGCAATGATATGGTGGAAACGACGACAGAATATTCTTCGGGCACAGCAGATTGCAG---------------------------------------------------------------------------  1753

                      *        2960         *        2980         *        3000         *        3020         *        3040         *        3060         *
Genomic-Seq : AATATTGCAGCAAGAGGAGCTCCTTATGCGAAAAAGAGAACACACGTATCTCATGACATCCAAATGTCACGGCACGGGCACAATAACCATGGCCAAGCTCGTACCGCTGTGGAAAATGGACCGAGCTTGTTGTCTTGA  3078
NM_102600   : ----------CAAGAGGAGCTCCTTATGCGAAAAAGAGAACACACGTATCTCATGACATCCAAATGTCACGGCACGGGCACAATAACCATGGCCAAGCTCGTACCGCTGTGGAAAATGGACCGAGCTTGTTGTCTTGA  1881
Cloned      : ----------CAAGAGGAGCTCCTTATGCGAAAAAGAGAACACACGTATCTCATGACATCCAAATGTCACGGCACGGGCACAATAACCATGGCCAAGCTCGTACCGCTGTGGAAAATGGACCGAGCTTGTTGTCTTGA  1881
```

AtRLP13

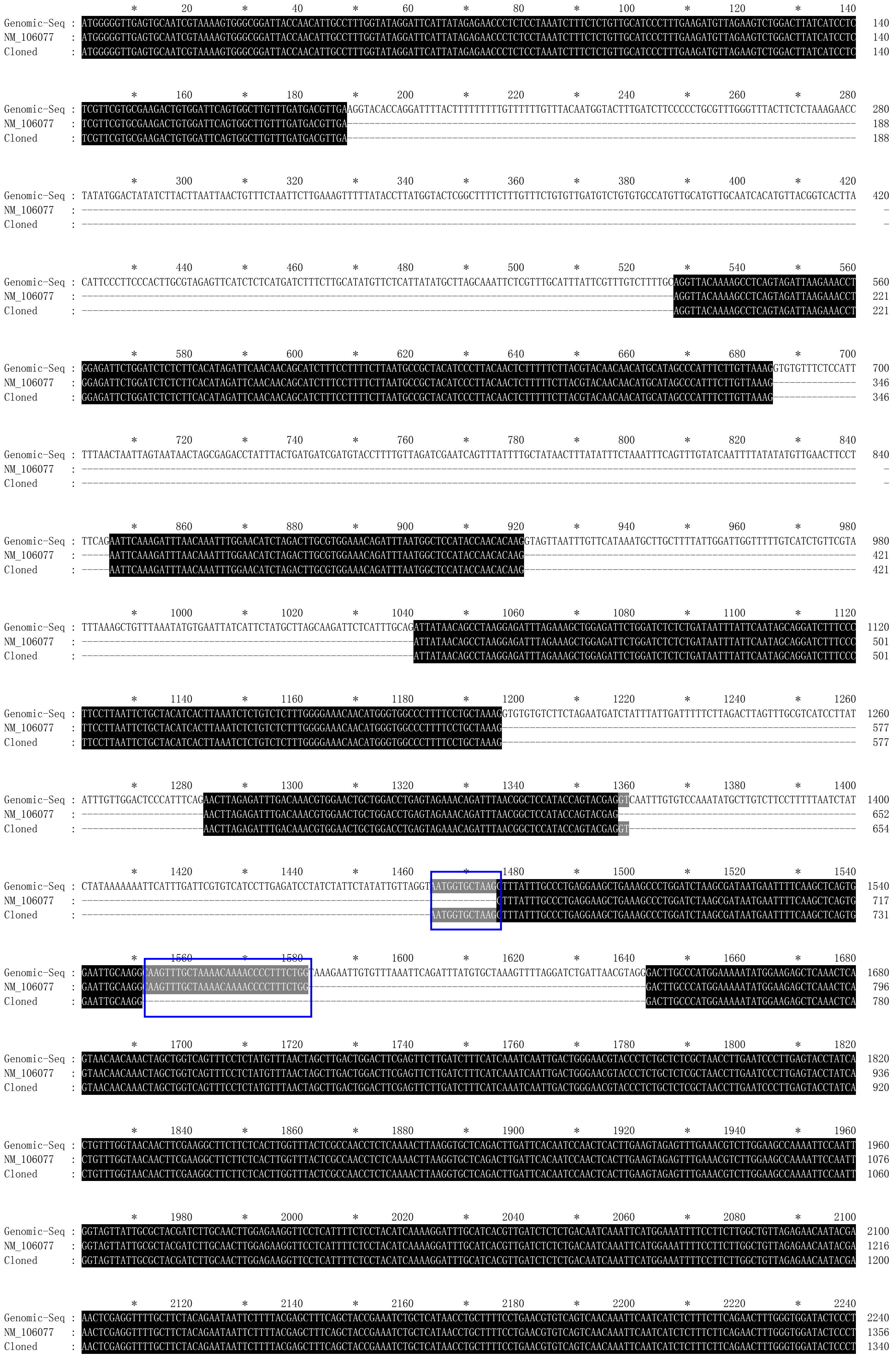

```
               *      2260        *      2280        *      2300        *      2320        *      2340        *      2360        *      2380
Genomic-Seq : CATTTAGTCTGTGTGAATTTAGCCTATAATGGTTTTCAAGGAAATCTGCCATCTTCTCTGGATAACATGAAGAGTATTGAATTTCTAGATTTGTCTCACAACAGGTTCCACGGGAAGCTACCAAGAAGATTTTTAAAGGG  2380
NM_106077   : CATTTAGTCTGTGTGAATTTAGCCTATAATGGTTTTCAAGGAAATCTGCCATCTTCTCTGGATAACATGAAGAGTATTGAATTTCTAGATTTGTCTCACAACAGGTTCCACGGGAAGCTACCAAGAAGATTTTTAAAGGG  1496
Cloned      : CATTTAGTCTGTGTGAATTTAGCCTATAATGGTTTTCAAGGAAATCTGCCATCTTCTCTGGATAACATGAAGAGTATTGAATTTCTAGATTTGTCTCACAACAGGTTCCACGGGAAGCTACCAAGAAGATTTTTAAAGGG  1480
```

```
               *      2400        *      2420        *      2440        *      2460        *      2480        *      2500        *      2520
Genomic-Seq : TTGTTATAACTTAACAATCTTGAAGCTGTCACACAACAAACTAAGTGGAGAGGTTTTTCCAGAAGCAGCCAACTTTACTCGTTTATGGGTGATGTCTATGGATAACAATCTGTTTACAGGAAATATTGGAAAAGGTTTTC  2520
NM_106077   : TTGTTATAACTTAACAATCTTGAAGCTGTCACACAACAAACTAAGTGGAGAGGTTTTTCCAGAAGCAGCCAACTTTACTCGTTTATGGGTGATGTCTATGGATAACAATCTGTTTACAGGAAATATTGGAAAAGGTTTTC  1636
Cloned      : TTGTTATAACTTAACAATCTTGAAGCTGTCACACAACAAACTAAGTGGAGAGGTTTTTCCAGAAGCAGCCAACTTTACTCGTTTATGGGTGATGTCTATGGATAACAATCTGTTTACAGGAAATATTGGAAAAGGTTTTC  1620
```

```
               *      2540        *      2560        *      2580        *      2600        *      2620        *      2640        *      2660
Genomic-Seq : GGAGCCTACCATCTTTGAATGTACTTGACATTTCAAACAACAAACTCACAGGTGTTATTCCAAGTTGGATTGGCGAACGTCAGGGATTATTCGCACTGCAGCTTTCAAACAACATGTTGGAAGGTGAAATACCTACTTCC  2660
NM_106077   : GGAGCCTACCATCTTTGAATGTACTTGACATTTCAAACAACAAACTCACAGGTGTTATTCCAAGTTGGATTGGCGAACGTCAGGGATTATTCGCACTGCAGCTTTCAAACAACATGTTGGAAGGTGAAATACCTACTTCC  1776
Cloned      : GGAGCCTACCATCTTTGAATGTACTTGACATTTCAAACAACAAACTCACAGGTGTTATTCCAAGTTGGATTGGCGAACGTCAGGGATTATTCGCACTGCAGCTTTCAAACAACATGTTGGAAGGTGAAATACCTACTTCC  1760
```

```
               *      2680        *      2700        *      2720        *      2740        *      2760        *      2780        *      2800
Genomic-Seq : TTGTTCAACATATCCTATCTTCAGCTACTGGACCTCTCTTCAAACAGGTTATCTGGGGATATACCTCCGCACGTAAGTTCCATATATCATGGGGCAGTGTTACTCCTCCAAAACAATAATTTATCAGGGGTTATTCCAGA  2800
NM_106077   : TTGTTCAACATATCCTATCTTCAGCTACTGGACCTCTCTTCAAACAGGTTATCTGGGGATATACCTCCGCACGTAAGTTCCATATATCATGGGGCAGTGTTACTCCTCCAAAACAATAATTTATCAGGGGTTATTCCAGA  1916
Cloned      : TTGTTCAACATATCCTATCTTCAGCTACTGGACCTCTCTTCAAACAGGTTATCTGGGGATATACCTCCGCACGTAAGTTCCATATATCATGGGGCAGTGTTACTCCTCCAAAACAATAATTTATCAGGGGTTATTCCAGA  1900
```

```
               *      2820        *      2840        *      2860        *      2880        *      2900        *      2920        *      2940
Genomic-Seq : CACACTACTACTGAATGTTATTGTACTTGATCTGAGAAATAACCGACTGTCTGGGAATCTTCCGGAGTTCATCAACACCCAAAACATCAGTATTCTTCTTTTGCGGGGGAACAATTTTACAGGACAGATTCCTCACCAGT  2940
NM_106077   : CACACTACTACTGAATGTTATTGTACTTGATCTGAGAAATAACCGACTGTCTGGGAATCTTCCGGAGTTCATCAACACCCAAAACATCAGTATTCTTCTTTTGCGGGGGAACAATTTTACAGGACAGATTCCTCACCAGT  2056
Cloned      : CACACTACTACTGAATGTTATTGTACTTGATCTGAGAAATAACCGACTGTCTGGGAATCTTCCGGAGTTCATCAACACCCAAAACATCAGTATTCTTCTTTTGCGGGGGAACAATTTTACAGGACAGATTCCTCACCAGT  2040
```

```
               *      2960        *      2980        *      3000        *      3020        *      3040        *      3060        *      3080
Genomic-Seq : TCTGCAGTCTAAGTAACATCCAACTTCTGGATCTTTCCAACAACAAATTCAATGGATCGATACCATCATGTCTTAGCAATACATCATTTGGTTTGAGGAAAGGGGATGATTCTTATAGATATGATGTCCCTTCAAGATTT  3080
NM_106077   : TCTGCAGTCTAAGTAACATCCAACTTCTGGATCTTTCCAACAACAAATTCAATGGATCGATACCATCATGTCTTAGCAATACATCATTTGGTTTGAGGAAAGGGGATGATTCTTATAGATATGATGTCCCTTCAAGATTT  2196
Cloned      : TCTGCAGTCTAAGTAACATCCAACTTCTGGATCTTTCCAACAACAAATTCAATGGATCGATACCATCATGTCTTAGCAATACATCATTTGGTTTGAGGAAAGGGGATGATTCTTATAGATATGATGTCCCTTCAAGATTT  2180
```

```
               *      3100        *      3120        *      3140        *      3160        *      3180        *      3200        *      3220
Genomic-Seq : GGCACAGCTAAGGATCCTGTATATTTTGAATCTCTACTCATGATAGATGAATTTAATATGGTCAATGAAACCAACTCCCAGACGAAAATTGAATTTGCAACAAAGCACAGGTATGATGCCTACATGGGTGGAAATCTCAA  3220
NM_106077   : GGCACAGCTAAGGATCCTGTATATTTTGAATCTCTACTCATGATAGATGAATTTAATATGGTCAATGAAACCAACTCCCAGACGAAAATTGAATTTGCAACAAAGCACAGGTATGATGCCTACATGGGTGGAAATCTCAA  2336
Cloned      : GGCACAGCTAAGGATCCTGTATATTTTGAATCTCTACTCATGATAGATGAATTTAATATGGTCAATGAAACCAACTCCCAGACGAAAATTGAATTTGCAACAAAGCACAGGTATGATGCCTACATGGGTGGAAATCTCAA  2320
```

```
               *      3240        *      3260        *      3280        *      3300        *      3320        *      3340        *      3360
Genomic-Seq : ATTATTGTTTGGAATGGATCTCTCAGAAAATGAGCTGAGTGGTGAGATCCCGGTAGAGCTTGGAGGTCTTGTGGAATTAGAAGCTCTCAATCTTTCTCACAACAACTTATCAGGAGTGATACTAGAAAGCTTTTCAGGTC  3360
NM_106077   : ATTATTGTTTGGAATGGATCTCTCAGAAAATGAGCTGAGTGGTGAGATCCCGGTAGAGCTTGGAGGTCTTGTGGAATTAGAAGCTCTCAATCTTTCTCACAACAACTTATCAGGAGTGATACTAGAAAGCTTTTCAGGTC  2476
Cloned      : ATTATTGTTTGGAATGGATCTCTCAGAAAATGAGCTGAGTGGTGAGATCCCGGTAGAGCTTGGAGGTCTTGTGGAATTAGAAGCTCTCAATCTTTCTCACAACAACTTATCAGGAGTGATACTAGAAAGCTTTTCAGGTC  2460
```

```
               *      3380        *      3400        *      3420        *      3440        *      3460        *      3480        *      3500
Genomic-Seq : TGAAGAATGTGGAAAGCCTTGATCTTTCTTTCAACAGATTACAAGGTCCTATCCCACTACAACTTACAGATATGATCAGCCTTGCTGTGTTCAATGTCTCCTACAACAACTTATCAGGAATCGTTCCACAGGGAAGACAG  3500
NM_106077   : TGAAGAATGTGGAAAGCCTTGATCTTTCTTTCAACAGATTACAAGGTCCTATCCCACTACAACTTACAGATATGATCAGCCTTGCTGTGTTCAATGTCTCCTACAACAACTTATCAGGAATCGTTCCACAGGGAAGACAG  2616
Cloned      : TGAAGAATGTGGAAAGCCTTGATCTTTCTTTCAACAGATTACAAGGTCCTATCCCACTACAACTTACAGATATGATCAGCCTTGCTGTGTTCAATGTCTCCTACAACAACTTATCAGGAATCGTTCCACAGGGAAGACAG  2600
```

```
               *      3520        *      3540        *      3560        *      3580        *      3600        *      3620        *      3640
Genomic-Seq : TTTAACACCTTTGAGACACAAAGCTACTTTGGTAATCCTCTTCTCTGTGGGAAGTCCATAGACATAAGCTGCGCGAGCAATAACTTTCACCCAACAGATAATGGAGTGGAAGCTGATGAATCTACAGTCGACATGGAATC  3640
NM_106077   : TTTAACACCTTTGAGACACAAAGCTACTTTGGTAATCCTCTTCTCTGTGGGAAGTCCATAGACATAAGCTGCGCGAGCAATAACTTTCACCCAACAGATAATGGAGTGGAAGCTGATGAATCTACAGTCGACATGGAATC  2756
Cloned      : TTTAACACCTTTGAGACACAAAGCTACTTTGGTAATCCTCTTCTCTGTGGGAAGTCCATAGACATAAGCTGCGCGAGCAATAACTTTCACCCAACAGATAATGGAGTGGAAGCTGATGAATCTACAGTCGACATGGAATC  2740
```

```
               *      3660        *      3680        *      3700        *      3720        *      3740        *      3760        *      3780
Genomic-Seq : TTTCTACTGGAGTTTTGTTGCAGCCTATGTGACAATACTTCTTGGAATACTTGCATCTCTCTCATTTGATTCTCCTTGGAGCAGAGCTTGGTTCTACATCGTTGATGCTTTCGTCCTCAAGGTTAGGAATATGTTGTGGC  3780
NM_106077   : TTTCTACTGGAGTTTTGTTGCAGCCTATGTGACAATACTTCTTGGAATACTTGCATCTCTCTCATTTGATTCTCCTTGGAGCAGAGCTTGGTTCTACATCGTTGATGCTTTCGTCCTCAAGGTTAGGAATATGTTGTGGC  2896
Cloned      : TTTCTACTGGAGTTTTGTTGCAGCCTATGTGACAATACTTCTTGGAATACTTGCATCTCTCTCATTTGATTCTCCTTGGAGCAGAGCTTGGTTCTACATCGTTGATGCTTTCGTCCTCAAGGTTAGGAATATGTTGTGGC  2880
```

```
               *      3800        *      3820        *      3840        *      3860        *      3880
Genomic-Seq : AAAACACTGCAGGTACCAAATGTTCTTACGTGCTAGTTTCATTGTCGTCCCCATCTGTTGTTGTTCTTCTCAAACCTCCTGCTTTGTTTCACAAAACCAGAACATAA  3887
NM_106077   : AAAACACTGCAGGTACCAAATGTTCTTACGTGCTAGTTTCATTGTCGTCCCCATCTGTTGTTGTTCTTCTCAAACCTCCTGCTTTGTTTCACAAAACCAGAACATAA  3003
Cloned      : AAAACACTGCAGGTACCAAATGTTCTTACGTGCTAGTTTCATTGTCGTCCCCATCTGTTGTTGTTCTTCTCAAACCTCCTGCTTTGTTTCACAAAACCAGAACATAA  2987
```

AtRLP20

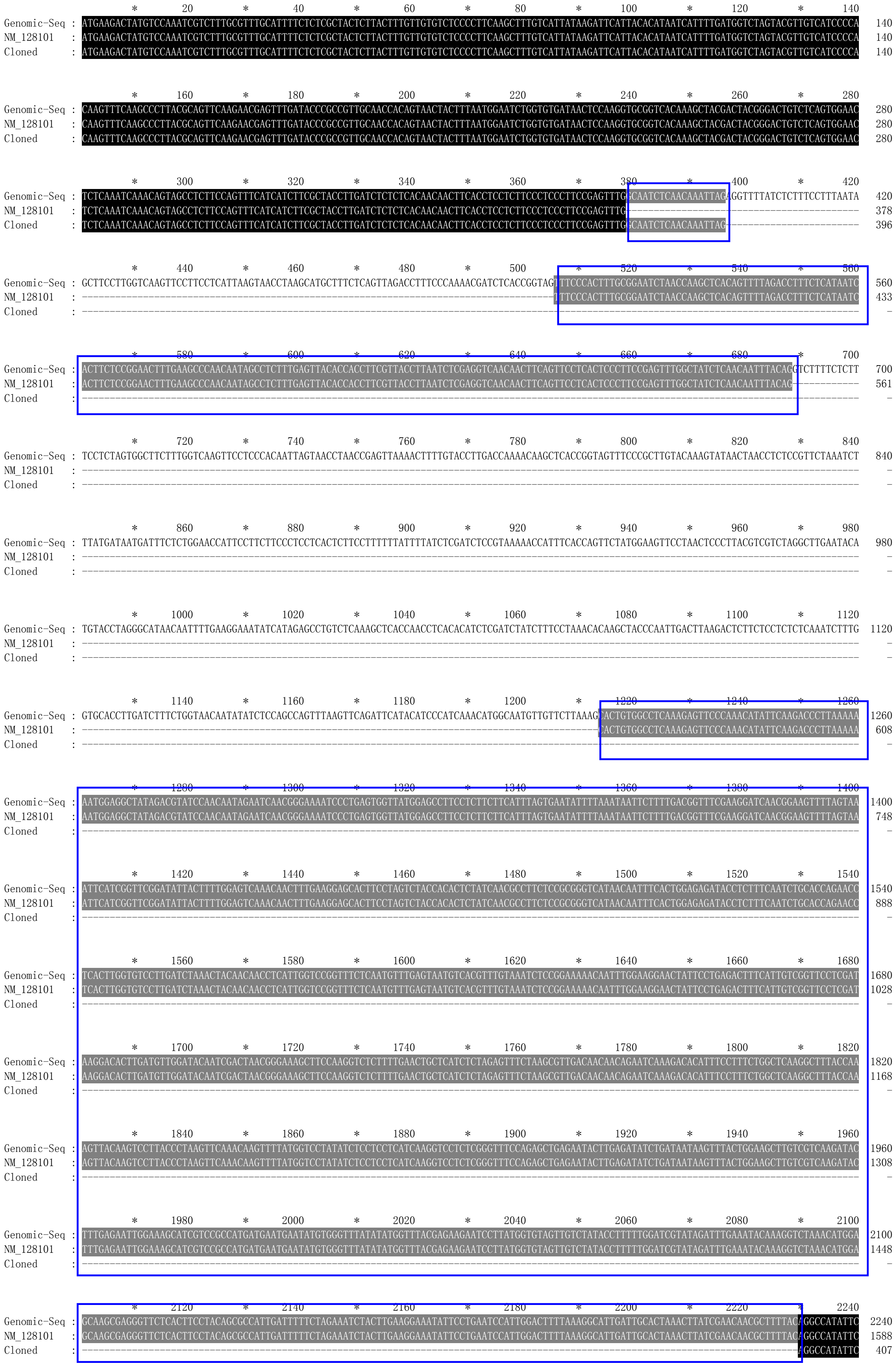

```
                        *       2260          *       2280          *       2300          *       2320          *       2340          *       2360          *       2380
Genomic-Seq : CTCAGTCTTTGGCAAATCTTAAGGAGCTCCAGTCACTAGACATGTCTAGGAACCAACTCTCAGGGACTATTCCTAATGGACTCAAGCAACTCTCGTTTTTGGCTTACATAAGTGTGTCTCATAACCAACTCAAGGGTGAA : 2380
NM_128101   : CTCAGTCTTTGGCAAATCTTAAGGAGCTCCAGTCACTAGACATGTCTAGGAACCAACTCTCAGGGACTATTCCTAATGGACTCAAGCAACTCTCGTTTTTGGCTTACATAAGTGTGTCTCATAACCAACTCAAGGGTGAA : 1728
Cloned      : CTCAGTCTTTGGCAAATCTTAAGGAGCTCCAGTCACTAGACATGTCTAGGAACCAACTCTCAGGGACTATTCCTAATGGACTCAAGCAACTCTCGTTTTTGGCTTACATAAGTGTGTCTCATAACCAACTCAAGGGTGAA :  547

                        *       2400          *       2420          *       2440          *       2460          *       2480          *       2500          *       2520
Genomic-Seq : ATACCACAAGGAACACAAATTACTGGGCAATTGAAATCTTCCTTTGAAGGGAATGTAGGACTTTGTGGTCTTCCTCTCGAGGAAAGGTGCTTCGACAATAGTGCATCTCCAACGCAGCACCACAAGCAAGACGAAGAAGA : 2520
NM_128101   : ATACCACAAGGAACACAAATTACTGGGCAATTGAAATCTTCCTTTGAAGGGAATGTAGGACTTTGTGGTCTTCCTCTCGAGGAAAGGTGCTTCGACAATAGTGCATCTCCAACGCAGCACCACAAGCAAGACGAAGAAGA : 1868
Cloned      : ATACCACAAGGAACACAAATTACTGGGCAATTGAAATCTTCCTTTGAAGGGAATGTAGGACTTTGTGGTCTTCCTCTCGAGGAAAGGTGCTTCGACAATAGTGCATCTCCAACGCAGCACCACAAGCAAGACGAAGAAGA :  687

                        *       2540          *       2560          *       2580          *       2600          *       2620          *       2640          *       2660
Genomic-Seq : AGAAGAAGAACAAGTGTTACACTGGAAAGCGGTGGCAATGGGGTATGGACCTGGATTGTTGGTTGGATTTGCAATTGCATATGTCATTGCTTCATACAAGCCGGAGTGGCTAACCAAGATAATTGGTCCGAATAAGCGCA : 2660
NM_128101   : AGAAGAAGAACAAGTGTTACACTGGAAAGCGGTGGCAATGGGGTATGGACCTGGATTGTTGGTTGGATTTGCAATTGCATATGTCATTGCTTCATACAAGCCGGAGTGGCTAACCAAGATAATTGGTCCGAATAAGCGCA : 2008
Cloned      : AGAAGAAGAACAAGTGTTACACTGGAAAGCGGTGGCAATGGGGTATGGACCTGGATTGTTGGTTGGATTTGCAATTGCATATGTCATTGCTTCATACAAGCCGGAGTGGCTAACCAAGATAATTGGTCCGAATAAGCGCA :  827

Genomic-Seq : GAAACTAG : 2668
NM_128101   : GAAACTAG : 2016
Cloned      : GAAACTAG :  835
```

AtRLP24

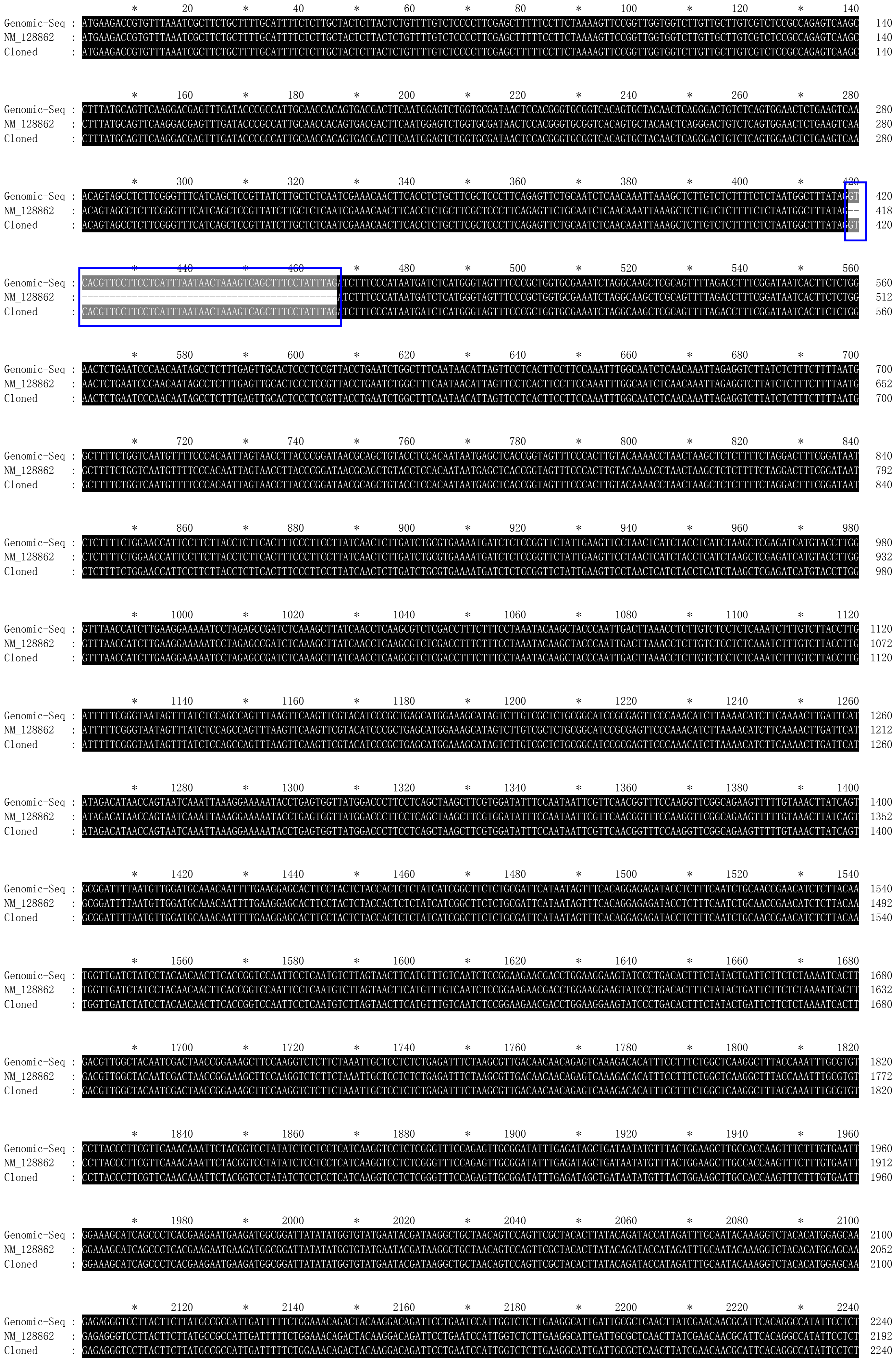

```
                  *     2260        *     2280        *     2300        *     2320        *     2340        *     2360        *     2380
Genomic-Seq : GTCTTTCGCCAATCTTATGAATCTCGAGTCACTAGACATGTCAGGAAACCAACTCTCTGGGACTATTCCTAATGGACTTGGGAGCCTCTCGTTTTTGGTGTACATAAGTGTGGCTCATAACAAACTCAAAGGTGAAATAC  2380
NM_128862   : GTCTTTCGCCAATCTTATGAATCTCGAGTCACTAGACATGTCAGGAAACCAACTCTCTGGGACTATTCCTAATGGACTTGGGAGCCTCTCGTTTTTGGTGTACATAAGTGTGGCTCATAACAAACTCAAAGGTGAAATAC  2332
Cloned      : GTCTTTCGCCAATCTTATGAATCTCGAGTCACTAGACATGTCAGGAAACCAACTCTCTGGGACTATTCCTAATGGACTTGGGAGCCTCTCGTTTTTGGTGTACATAAGTGTGGCTCATAACAAACTCAAAGGTGAAATAC  2380

                  *     2400        *     2420        *     2440        *     2460        *     2480        *     2500        *     2520
Genomic-Seq : CACAAGGAACACAAATTACCGGGCAAATTAAATCATCTTTCGAAGGGAATGCAGGGCTTTGTGGTCTTCCTCTCCAGGAAACTTGCTTTGACTCTAGTGTGCCACCGATACAACCAAAGCAAGAAGACGAAGAAAAAGGA  2520
NM_128862   : CACAAGGAACACAAATTACCGGGCAAATTAAATCATCTTTCGAAGGGAATGCAGGGCTTTGTGGTCTTCCTCTCCAGGAAACTTGCTTTGACTCTAGTGTGCCACCGATACAACCAAAGCAAGAAGACGAAGAAAAAGGA  2472
Cloned      : CACAAGGAACACAAATTACCGGGCAAATTAAATCATCTTTCGAAGGGAATGCAGGGCTTTGTGGTCTTCCTCTCCAGGAAACTTGCTTTGACTCTAGTGTGCCACCGATACAACCAAAGCAAGAAGACGAAGAAAAAGGA  2520

                  *     2540        *     2560        *     2580        *     2600        *     2620        *     2640
Genomic-Seq : GAGGTGATTAACTGGAAAGCAGTAGCTATAGGATATGCTCCTGGATTGTTGTTTGGATTGGCAATAGCACATCTCATTGCTTCATACAAGCCAGAGTGGCTCGTCAAAATTATTGGCTTCTGA  2643
NM_128862   : GAGGTGATTAACTGGAAAGCAGTAGCTATAGGATATGCTCCTGGATTGTTGTTTGGATTGGCAATAGCACATCTCATTGCTTCATACAAGCCAGAGTGGCTCGTCAAAATTATTGGCTTCTGA  2595
Cloned      : GAGGTGATTAACTGGAAAGCAGTAGCTATAGGATATGCTCCTGGATTGTTGTTTGGATTGGCAATAGCACATCTCATTGCTTCATACAAGCCAGAGTGGCTCGTCAAAATTATTGGCTTCTGA  2643
```

AtRLP40

```
                  *        20         *        40         *        60         *        80         *       100         *       120         *       140
Genomic-Seq : ATGCAAAACCACATTCTATCTTTATTTATACCAATCTTCTCTCTCACATTTCTTATATCAAAATCCATTCTTTCTTTCAAAGCTGTTTTCATCATGTCTGAATTGCTTTTCAGTTTGAATTTTCTCTTGCTACTCTTACT : 140
NM_113404   : ATGCAAAACCACATTCTATCTTTATTTATACCAATCTTCTCTCTCACATTTCTTATATCAAAATCCATTCTTTCTTTCAAAGCTGTTTTCATCATGTCTGAATTGCTTTTCAGTTTGAATTTTCTCTTGCTACTCTTACT : 140
Cloned      : ATGCAAAACCACATTCTATCTTTATTTATACCAATCTTCTCTCTCACATTTCTTATATCAAAATCCATTCTTTCTTTCAAAGCTGTTTTCATCATGTCTGAATTGCTTTTCAGTTTGAATTTTCTCTTGCTACTCTTACT : 140

                  *       160         *       180         *       200         *       220         *       240         *       260         *       280
Genomic-Seq : ATCTTGTGTCTCTCCTTCAAGCTTCTTCACTTTTAATAACCCTGTTGTTGGTCTTGGTGCTTGTGGTCCCCACCAGATTCAAGCCTTTACGCAGTTCAAGAACGAGTTTGATACTCGTGCTTGCAACCATAGTGACCCTT : 280
NM_113404   : ATCTTGTGTCTCTCCTTCAAGCTTCTTCACTTTTAATAACCCTGTTGTTGGTCTTGGTGCTTGTGGTCCCCACCAGATTCAAGCCTTTACGCAGTTCAAGAACGAGTTTGATACTCGTGCTTGCAACCATAGTGACCCTT : 280
Cloned      : ATCTTGTGTCTCTCCTTCAAGCTTCTTCACTTTTAATAACCCTGTTGTTGGTCTTGGTGCTTGTGGTCCCCACCAGATTCAAGCCTTTACGCAGTTCAAGAACGAGTTTGATACTCGTGCTTGCAACCATAGTGACCCTT : 280

                  *       300         *       320         *       340         *       360         *       380         *       400         *       420
Genomic-Seq : GGAATGGAGTGTGGTGCGATGACTCCACGGGTGCGGTCACGATGCTACAACTCAGGGCATGTCTTAGCGGAACTCTGAAACCTAACAGTAGCCTTTTCCAGTTTCATCATCTCCGTTCCCTTCTTCTCCCTCATAACAAC : 420
NM_113404   : GGAATGGAGTGTGGTGCGATGACTCCACGGGTGCGGTCACGATGCTACAACTCAGGGCATGTCTTAGCGGAACTCTGAAACCTAACAGTAGCCTTTTCCAGTTTCATCATCTCCGTTCCCTTCTTCTCCCTCATAACAAC : 420
Cloned      : GGAATGGAGTGTGGTGCGATGACTCCACGGGTGCGGTCACGATGCTACAACTCAGGGCATGTCTTAGCGGAACTCTGAAACCTAACAGTAGCCTTTTCCAGTTTCATCATCTCCGTTCCCTTCTTCTCCCTCATAACAAC : 420

                  *       440         *       460         *       480         *       500         *       520         *       540         *       560
Genomic-Seq : TTCACCTCCTCTTCAATTTCTTCCAAGTTTGGAATGCTCAACAACTTAGAGGTATTGTCTCTTTCAAGTAGTGGCTTCCTCGCCCAAGTTCCTTTTTCATTTAGTAACCTAAGCATGCTTTCCGCCTTAGTCCTTTCCAA : 560
NM_113404   : TTCACCTCCTCTTCAATTTCTTCCAAGTTTGGAATGCTCAACAACTTAGAGGTATTGTCTCTTTCAAGTAGTGGCTTCCTCGCCCAAGTTCCTTTTTCATTTAGTAACCTAAGCATGCTTTCCGCCTTAGTCCTTTCCAA : 560
Cloned      : TTCACCTCCTCTTCAATTTCTTCCAAGTTTGGAATGCTCAACAACTTAGAGGTATTGTCTCTTTCAAGTAGTGGCTTCCTCGCCCAAGTTCCTTTTTCATTTAGTAACCTAAGCATGCTTTCCGCCTTAGTCCTTTCCAA : 560

                  *       580         *       600         *       620         *       640         *       660         *       680         *       700
Genomic-Seq : TAATGACCTCACTGGTAGTTTATCATTTGCGCGGAATCTACGCAAGCTCAGAGTTTTAGATGTTTCTTATAATCACTTCTCTGGAATTTTGAATCCCAATAGTAGCCTCTTTGAGTTGCACCACATCATTTACCTTAATC : 700
NM_113404   : TAATGACCTCACTGGTAGTTTATCATTTGCGCGGAATCTACGCAAGCTCAGAGTTTTAGATGTTTCTTATAATCACTTCTCTGGAATTTTGAATCCCAATAGTAGCCTCTTTGAGTTGCACCACATCATTTACCTTAATC : 700
Cloned      : TAATGACCTCACTGGTAGTTTATCATTTGCGCGGAATCTACGCAAGCTCAGAGTTTTAGATGTTTCTTATAATCACTTCTCTGGAATTTTGAATCCCAATAGTAGCCTCTTTGAGTTGCACCACATCATTTACCTTAATC : 700

                  *       720         *       740         *       760         *       780         *       800         *       820         *       840
Genomic-Seq : TCCGTTACAACAACTTCACCTCCTCTTCACTCCCTTATGAATTTGGCAATCTCAACAAACTAGAGGTCTTGGATGTTTCCTCTAATAGCTTCTTCGGTCAAGTTCCTCCCACTATTAGTAACCTAACCCAGTTAACCGAG : 840
NM_113404   : TCCGTTACAACAACTTCACCTCCTCTTCACTCCCTTATGAATTTGGCAATCTCAACAAACTAGAGGTCTTGGATGTTTCCTCTAATAGCTTCTTCGGTCAAGTTCCTCCCACTATTAGTAACCTAACCCAGTTAACCGAG : 840
Cloned      : TCCGTTACAACAACTTCACCTCCTCTTCACTCCCTTATGAATTTGGCAATCTCAACAAACTAGAGGTCTTGGATGTTTCCTCTAATAGCTTCTTCGGTCAAGTTCCTCCCACTATTAGTAACCTAACCCAGTTAACCGAG : 840

                  *       860         *       880         *       900         *       920         *       940         *       960         *       980
Genomic-Seq : TTGTACCTTCCCTTGAACCACTTCACTGGTAGTCTTCCGCTTGTACAAAATCTAACCAAGCTCTCCATTCTACATCTTTTTGGTAATCACTTCTCTGGAACAATCCCTTCTTCTCTCTTCACTATGCCTTTCTTATCCTA : 980
NM_113404   : TTGTACCTTCCCTTGAACCACTTCACTGGTAGTCTTCCGCTTGTACAAAATCTAACCAAGCTCTCCATTCTACATCTTTTTGGTAATCACTTCTCTGGAACAATCCCTTCTTCTCTCTTCACTATGCCTTTCTTATCCTA : 980
Cloned      : TTGTACCTTCCCTTGAACCACTTCACTGGTAGTCTTCCGCTTGTACAAAATCTAACCAAGCTCTCCATTCTACATCTTTTTGGTAATCACTTCTCTGGAACAATCCCTTCTTCTCTCTTCACTATGCCTTTCTTATCCTA : 980

                  *      1000         *      1020         *      1040         *      1060         *      1080         *      1100         *      1120
Genomic-Seq : TCTTTCATTAAAAGGAAACAATCTCAACGGTTCAATTGAAGTTCCTAACTCATCTTCGTCATCAAGGCTAGAGAGTTTGCACCTAGGTGAAAACCATTTCGAAGGAAAAATCCTAGAGCCTATCTCAAAGCTCATCAACC : 1120
NM_113404   : TCTTTCATTAAAAGGAAACAATCTCAACGGTTCAATTGAAGTTCCTAACTCATCTTCGTCATCAAGGCTAGAGAGTTTGCACCTAGGTGAAAACCATTTCGAAGGAAAAATCCTAGAGCCTATCTCAAAGCTCATCAACC : 1120
Cloned      : TCTTTCATTAAAAGGAAACAATCTCAACGGTTCAATTGAAGTTCCTAACTCATCTTCGTCATCAAGGCTAGAGAGTTTGCACCTAGGTGAAAACCATTTCGAAGGAAAAATCCTAGAGCCTATCTCAAAGCTCATCAACC : 1120

                  *      1140         *      1160         *      1180         *      1200         *      1220         *      1240         *      1260
Genomic-Seq : TCAAAGAGCTCGACCTTTCTTTCCTAAACACAAGCTACCCAATTGACTTAAGTCTCTTCTCCTCTCTCAAATCTTTGTTGCTCCTCGATCTTTCCGGTGATTGGATATCAAAGGCTAGTTTAACTTTGGATTCATACATT : 1260
NM_113404   : TCAAAGAGCTCGACCTTTCTTTCCTAAACACAAGCTACCCAATTGACTTAAGTCTCTTCTCCTCTCTCAAATCTTTGTTGCTCCTCGATCTTTCCGGTGATTGGATATCAAAGGCTAGTTTAACTTTGGATTCATACATT : 1260
Cloned      : TCAAAGAGCTCGACCTTTCTTTCCTAAACACAAGCTACCCAATTGACTTAAGTCTCTTCTCCTCTCTCAAATCTTTGTTGCTCCTCGATCTTTCCGGTGATTGGATATCAAAGGCTAGTTTAACTTTGGATTCATACATT : 1260

                  *      1280         *      1300         *      1320         *      1340         *      1360         *      1380         *      1400
Genomic-Seq : CCATCTACCCTGGAAGTGTTGCGTTTGGAGCATTGCGACATCAGTGATTTCCCTAACGTCTTCAAGACCCTTCATAATTTGGAGTATATTGCCTTATCCAACAATAGAATCAGTGGGAAATTTCCAGAGTGGTTATGGAG : 1400
NM_113404   : CCATCTACCCTGGAAGTGTTGCGTTTGGAGCATTGCGACATCAGTGATTTCCCTAACGTCTTCAAGACCCTTCATAATTTGGAGTATATTGCCTTATCCAACAATAGAATCAGTGGGAAATTTCCAGAGTGGTTATGGAG : 1400
Cloned      : CCATCTACCCTGGAAGTGTTGCGTTTGGAGCATTGCGACATCAGTGATTTCCCTAACGTCTTCAAGACCCTTCATAATTTGGAGTATATTGCCTTATCCAACAATAGAATCAGTGGGAAATTTCCAGAGTGGTTATGGAG : 1400

                  *      1420         *      1440         *      1460         *      1480         *      1500         *      1520         *      1540
Genomic-Seq : CCTTCCTCGTCTGAGCTCAGTGTTCATTACAGATAATTTGTTAACTGGCTTTGAAGGGTCATCAGAAGTTTTAGTAAATTCATCAGTGCAGATCTTAAGTTTGGATACAAACAGTTTAGAAGGGGCACTCCCGCATCTAC : 1540
NM_113404   : CCTTCCTCGTCTGAGCTCAGTGTTCATTACAGATAATTTGTTAACTGGCTTTGAAGGGTCATCAGAAGTTTTAGTAAATTCATCAGTGCAGATCTTAAGTTTGGATACAAACAGTTTAGAAGGGGCACTCCCGCATCTAC : 1540
Cloned      : CCTTCCTCGTCTGAGCTCAGTGTTCATTACAGATAATTTGTTAACTGGCTTTGAAGGGTCATCAGAAGTTTTAGTAAATTCATCAGTGCAGATCTTAAGTTTGGATACAAACAGTTTAGAAGGGGCACTCCCGCATCTAC : 1540

                  *      1560         *      1580         *      1600         *      1620         *      1640         *      1660         *      1680
Genomic-Seq : CGCTCTCTATCAACTATTTCTCTGCAATAGACAATAGATTCGGAGGCGACATACCTCTTTCAATCTGTAATAGAAGCTCGCTTGATGTTCTTGATCTAAGCTACAACAACTTCACCGGACCAATTCCTCCATGTCTGAGT : 1680
NM_113404   : CGCTCTCTATCAACTATTTCTCTGCAATAGACAATAGATTCGGAGGCGACATACCTCTTTCAATCTGTAATAGAAGCTCGCTTGATGTTCTTGATCTAAGCTACAACAACTTCACCGGACCAATTCCTCCATGTCTGAGT : 1680
Cloned      : CGCTCTCTATCAACTATTTCTCTGCAATAGACAATAGATTCGGAGGCGACATACCTCTTTCAATCTGTAATAGAAGCTCGCTTGATGTTCTTGATCTAAGCTACAACAACTTCACCGGACCAATTCCTCCATGTCTGAGT : 1680

                  *      1700         *      1720         *      1740         *      1760         *      1780         *      1800         *      1820
Genomic-Seq : AACTTATTGTATTTGAAACTCCGAAAGAACAACTTGGAAGGAAGTATTCCTGACAAGTATTATGAGGATACGCCTCTACGGTCACTCGACGTTGGCTACAATCGATTAACAGGGAAGCTGCCAAGGTCTCTTATAAATTG : 1820
NM_113404   : AACTTATTGTATTTGAAACTCCGAAAGAACAACTTGGAAGGAAGTATTCCTGACAAGTATTATGAGGATACGCCTCTACGGTCACTCGACGTTGGCTACAATCGATTAACAGGGAAGCTGCCAAGGTCTCTTATAAATTG : 1820
Cloned      : AACTTATTGTATTTGAAACTCCGAAAGAACAACTTGGAAGGAAGTATTCCTGACAAGTATTATGAGGATACGCCTCTACGGTCACTCGACGTTGGCTACAATCGATTAACAGGGAAGCTGCCAAGGTCTCTTATAAATTG : 1820

                  *      1840         *      1860         *      1880         *      1900         *      1920         *      1940         *      1960
Genomic-Seq : CTCAGCTCTACAGTTTCTAAGTGTGGACCACAACGGAATCAAAGATACATTTCCTTTCTCCCTCAAGGCTTTACCGAAATTGCAAGTCCTTCTCCTCAGTTCAAACAAATTCTATGGTCCTCTATCTCCTCCTAATGAAG : 1960
NM_113404   : CTCAGCTCTACAGTTTCTAAGTGTGGACCACAACGGAATCAAAGATACATTTCCTTTCTCCCTCAAGGCTTTACCGAAATTGCAAGTCCTTCTCCTCAGTTCAAACAAATTCTATGGTCCTCTATCTCCTCCTAATGAAG : 1960
Cloned      : CTCAGCTCTACAGTTTCTAAGTGTGGACCACAACGGAATCAAAGATACATTTCCTTTCTCCCTCAAGGCTTTACCGAAATTGCAAGTCCTTCTCCTCAGTTCAAACAAATTCTATGGTCCTCTATCTCCTCCTAATGAAG : 1960

                  *      1980         *      2000         *      2020         *      2040         *      2060         *      2080         *      2100
Genomic-Seq : GTCCTCTGGGTTTTCCTGAGCTGCGGATACTGGAGATAGCTGGTAACAAACTAACCGGAAGCTTGTCGACGGCCGGCCGCGGCCGGCCGTCGACAAGCTTGTCCTCAGACTTTTTGTGAATTGGAAAGCATCATCACAC : 2100
NM_113404   : GTCCTCTGGGTTTTCCTGAGCTGCGGATACTGGAGATAGCTGGTAACAAACTAACCGGAAGCTT---------------------------------CTTGTCCTCAGACTTTTTGTGAATTGGAAAGCATCATCACAC : 2067
Cloned      : GTCCTCTGGGTTTTCCTGAGCTGCGGATACTGGAGATAGCTGGTAACAAACTAACCGGAAGCTT------------------------------------GTCCTCAGACTTTTTGTGAATTGGAAAGCATCATCACAC : 2064

                  *      2120         *      2140         *      2160         *      2180         *      2200         *      2220         *      2240
Genomic-Seq : ACGATGAATGAAGATCTGGGTCTATATATGGTATATGGCAAGGTTATTTTCGGGAACTATCACCTCACCTATTATGAAACTATAGATTTACGATATAAAGGTCTATCTATGGAGCAAAGGAACGTCCTTACTTCCTCAGC : 2240
NM_113404   : ACGATGAATGAAGATCTGGGTCTATATATGGTATATGGCAAGGTTATTTTCGGGAACTATCACCTCACCTATTATGAAACTATAGATTTACGATATAAAGGTCTATCTATGGAGCAAAGGAACGTCCTTACTTCCTCAGC : 2207
Cloned      : ACGATGAATGAAGATCTGGGTCTATATATGGTATATGGCAAGGTTATTTTCGGGAACTATCACCTCACCTATTATGAAACTATAGATTTACGATATAAAGGTCTATCTATGGAGCAAAGGAACGTCCTTACTTCCTCAGC : 2204
```

```
                     *        2260          *        2280          *        2300          *        2320          *        2340          *        2360          *        2380
Genomic-Seq : CACCATTGATTTTTCAGGAAACAGACTTGAAGGAGAAATTCCTGAATCTATTGGTCTCTTAAAGGCACTGATCGCACTCAACTTATCGAACAACGCCTTCACAGGCCATATTCCTCTTTCTTTCGCCAATCTTAAGAAGA  2380
NM_113404   : CACCATTGATTTTTCAGGAAACAGACTTGAAGGAGAAATTCCTGAATCTATTGGTCTCTTAAAGGCACTGATCGCACTCAACTTATCGAACAACGCCTTCACAGGCCATATTCCTCTTTCTTTCGCCAATCTTAAGAAGA  2347
Cloned      : CACCATTGATTTTTCAGGAAACAGACTTGAAGGAGAAATTCCTGAATCTATTGGTCTCTTAAAGGCACTGATCGCACTCAACTTATCGAACAACGCCTTCACAGGCCATATTCCTCTTTCTTTCGCCAATCTTAAGAAGA  2344

                     *        2400          *        2420          *        2440          *        2460          *        2480          *        2500          *        2520
Genomic-Seq : TGGAGTCACTAGACCTATCAAGTAACCAACTCTCAGGGACTATTCCTAATGGCCTAAGGACCCTCTCGTTTTTGGCATACGTGAACGTGTCTCACAACCAACTCATTGGTGAAATACCACAAGGAACACAAATAACTGGG  2520
NM_113404   : TGGAGTCACTAGACCTATCAAGTAACCAACTCTCAGGGACTATTCCTAATGGCCTAAGGACCCTCTCGTTTTTGGCATACGTGAACGTGTCTCACAACCAACTCATTGGTGAAATACCACAAGGAACACAAATAACTGGG  2487
Cloned      : TGGAGTCACTAGACCTATCAAGTAACCAACTCTCAGGGACTATTCCTAATGGCCTAAGGACCCTCTCGTTTTTGGCATACGTGAACGTGTCTCACAACCAACTCATTGGTGAAATACCACAAGGAACACAAATAACTGGG  2484

                     *        2540          *        2560          *        2580          *        2600          *        2620          *        2640          *        2660
Genomic-Seq : CAACCTAAATCGTCATTTGAAGGAAATGCAGGGCTTTGCGGTTTTCCTCTTCAAGAAAGTTGCTTCGGGACTAACACACCACCAGCACAACATCCTAAAGAACAAGAAGAAGAGGAAGAAGATGAGCAAGTGTTAAACTG  2660
NM_113404   : CAACCTAAATCGTCATTTGAAGGAAATGCAGGGCTTTGCGGTTTTCCTCTTCAAGAAAGTTGCTTCGGGACTAACACACCACCAGCACAACATCCTAAAGAACAAGAAGAAGAGGAAGAAGATGAGCAAGTGTTAAACTG  2627
Cloned      : CAACCTAAATCGTCATTTGAAGGAAATGCAGGGCTTTGCGGTTTTCCTCTTCAAGAAAGTTGCTTCGGGACTAACACACCACCAGCACAACATCCTAAAGAACAAGAAGAAGAGGAAGAAGATGAGCAAGTGTTAAACTG  2624

                     *        2680          *        2700          *        2720          *        2740          *        2760          *        2780
Genomic-Seq : GAAAGCAGTGGCAATAGGGTACGGAATTGGAGTGTTACTTGGATTGGCAATAGCACAACTCATTTCTTTATATAAACCGAAGTGGCTAGCTTCTCTGGTAATTAAGAGCAGAAACTGTTAA  2781
NM_113404   : GAAAGCAGTGGCAATAGGGTACGGAATTGGAGTGTTACTTGGATTGGCAATAGCACAACTCATTTCTTTATATAAACCGAAGTGGCTAGCTTCTCTGGTAATTAAGAGCAGAAACTGTTAA  2748
Cloned      : GAAAGCAGTGGCAATAGGGTACGGAATTGGAGTGTTACTTGGATTGGCAATAGCACAACTCATTTCTTTATATAAACCGAAGTGGCTAGCTTCTCTGGTAATTAAGAGCAGAAACTGTTAA  2745
```

AtRLP52

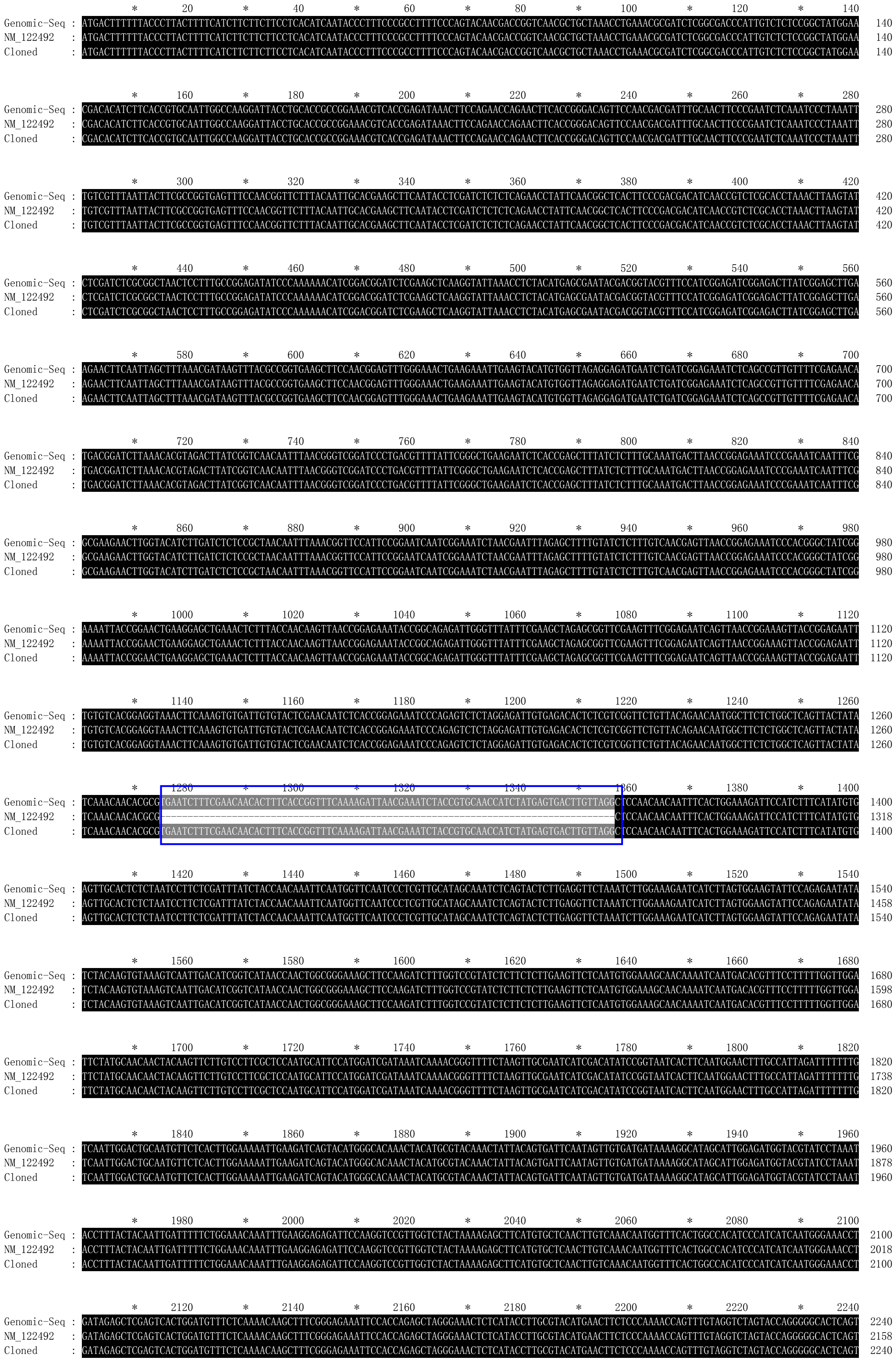

```
                     *       2260           *       2280           *       2300           *       2320           *       2340           *       2360           *       2380
Genomic-Seq : TTCAAACACAGCCTTGCTCTTCTTTTGCGGACAACCCGAGACTCTTTGGCCTTTCACTTGAAAGAGTTTGTGTAGATATCCACAAGAAAACACCACAACAATCCGAAATGCCAGAGCCTGAAGAAGATGAAGAAGAGGTG  2380
NM_122492   : TTCAAACACAGCCTTGCTCTTCTTTTGCGGACAACCCGAGACTCTTTGGCCTTTCACTTGAAAGAGTTTGTGTAGATATCCACAAGAAAACACCACAACAATCCGAAATGCCAGAGCCTGAAGAAGATGAAGAAGAGGTG  2298
Cloned      : TTCAAACACAGCCTTGCTCTTCTTTTGCGGACAACCCGAGACTCTTTGGCCTTTCACTTGAAAGAGTTTGTGTAGATATCCACAAGAAAACACCACAACAATCCGAAATGCCAGAGCCTGAAGAAGATGAAGAAGAGGTG  2380

                     *       2400           *       2420           *       2440           *       2460           *       2480           *       2500           *
Genomic-Seq : ATGAATTGGACAGCAGCTGCAATTGGATCCATACCTGGTATTTCCATTGGATTGACGATGGGATACATATTGGTTTCTTACAAACCAGAATGGTTAATGAACTCTGGCCGAAACAAACGCAGAATTAAACCAATATAA  2518
NM_122492   : ATGAATTGGACAGCAGCTGCAATTGGATCCATACCTGGTATTTCCATTGGATTGACGATGGGATACATATTGGTTTCTTACAAACCAGAATGGTTAATGAACTCTGGCCGAAACAAACGCAGAATTAAACCAATATAA  2436
Cloned      : ATGAATTGGACAGCAGCTGCAATTGGATCCATACCTGGTATTTCCATTGGATTGACGATGGGATACATATTGGTTTCTTACAAACCAGAATGGTTAATGAACTCTGGCCGAAACAAACGCAGAATTAAACCAATATAA  2518
```

AtRLP56

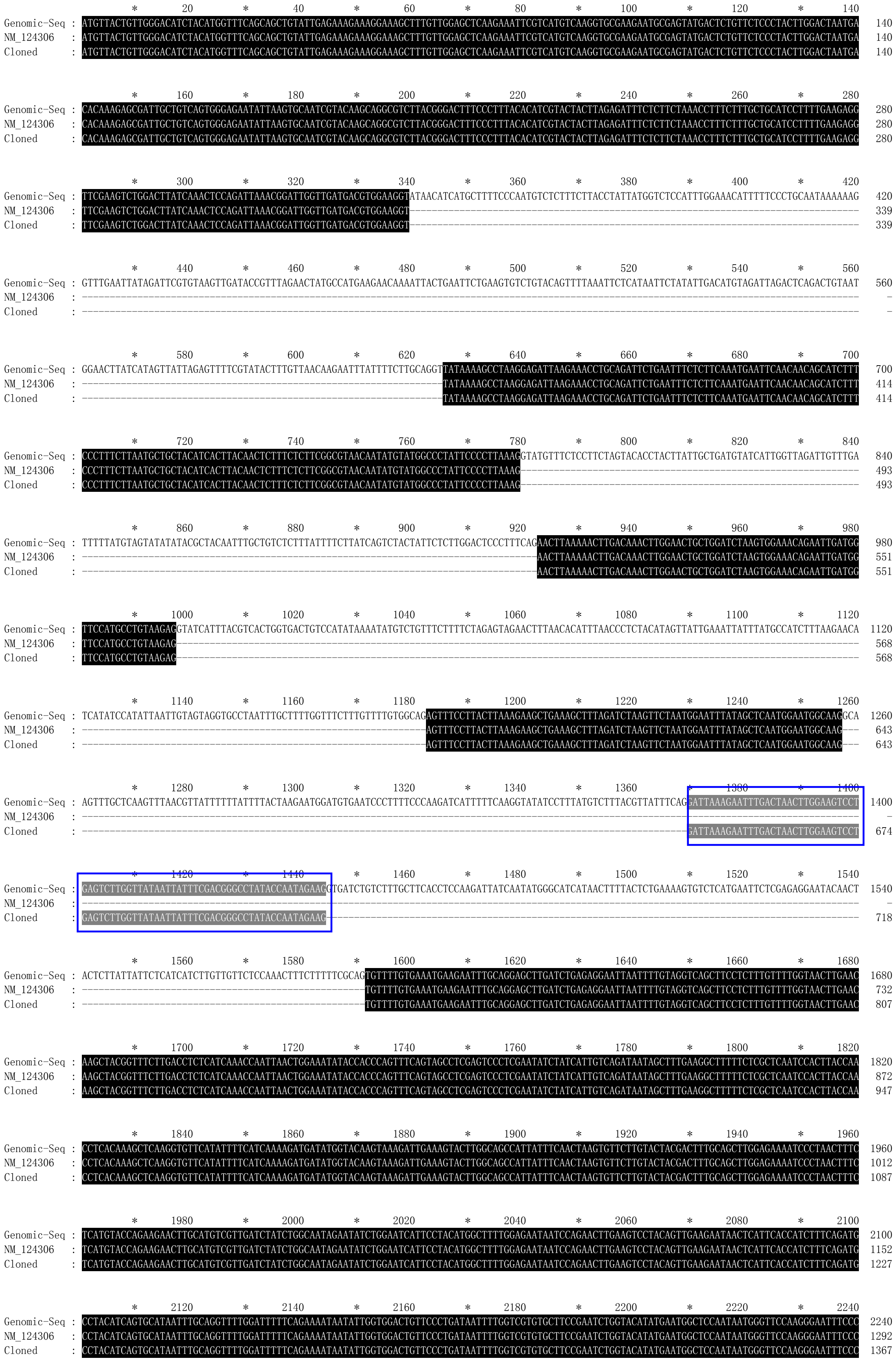

```
                 *     2260         *     2280         *     2300         *     2320         *     2340         *     2360         *     2380
Genomic-Seq : ATCCTCCATGGGTGAAATGTATAATATTTCATTCCTGGACCTGTCTTATAACAATTTATCAGGGGAGTTACCTCAAAGTTTTGTCTCAAGTTGTTTTTCATTAAGCATCCTGCAGCTGTCTCACAACAAATTTAGCGGCC  2380
NM_124306   : ATCCTCCATGGGTGAAATGTATAATATTTCATTCCTGGACCTGTCTTATAACAATTTATCAGGGGAGTTACCTCAAAGTTTTGTCTCAAGTTGTTTTTCATTAAGCATCCTGCAGCTGTCTCACAACAAATTTAGCGGCC  1432
Cloned      : ATCCTCCATGGGTGAAATGTATAATATTTCATTCCTGGACCTGTCTTATAACAATTTATCAGGGGAGTTACCTCAAAGTTTTGTCTCAAGTTGTTTTTCATTAAGCATCCTGCAGCTGTCTCACAACAAATTTAGCGGCC  1507

                 *     2400         *     2420         *     2440         *     2460         *     2480         *     2500         *     2520
Genomic-Seq : ATTTTCTTCCAAGACAAACAAACTTCACTTCATTGATCGTGCTGAGAATCAATAACAACTTATTTACTGGGAAGATCGGAGTTGGTTTGCTTACCTTGGTTGATTTGTGTATACTTGACATGTCCAACAATTTCTTAGAA  2520
NM_124306   : ATTTTCTTCCAAGACAAACAAACTTCACTTCATTGATCGTGCTGAGAATCAATAACAACTTATTTACTGGGAAGATCGGAGTTGGTTTGCTTACCTTGGTTGATTTGTGTATACTTGACATGTCCAACAATTTCTTAGAA  1572
Cloned      : ATTTTCTTCCAAGACAAACAAACTTCACTTCATTGATCGTGCTGAGAATCAATAACAACTTATTTACTGGGAAGATCGGAGTTGGTTTGCTTACCTTGGTTGATTTGTGTATACTTGACATGTCCAACAATTTCTTAGAA  1647

                 *     2540         *     2560         *     2580         *     2600         *     2620         *     2640         *     2660
Genomic-Seq : GGTGAGCTACCACCTTTGCTTCTGGTATTTGAATATCTTAATTTTCTGGACCTCTCTGGAAATCTATTATCTGGAGCCTTACCATCACATGTTAGTTTGGATAATGTTTTGTTCCTACACAACAACAACTTCACAGGGCC  2660
NM_124306   : GGTGAGCTACCACCTTTGCTTCTGGTATTTGAATATCTTAATTTTCTGGACCTCTCTGGAAATCTATTATCTGGAGCCTTACCATCACATGTTAGTTTGGATAATGTTTTGTTCCTACACAACAACAACTTCACAGGGCC  1712
Cloned      : GGTGAGCTACCACCTTTGCTTCTGGTATTTGAATATCTTAATTTTCTGGACCTCTCTGGAAATCTATTATCTGGAGCCTTACCATCACATGTTAGTTTGGATAATGTTTTGTTCCTACACAACAACAACTTCACAGGGCC  1787

                 *     2680         *     2700         *     2720         *     2740         *     2760         *     2780         *     2800
Genomic-Seq : AATTCCGGACACATTTTTGGGAAGCATCCAGATACTTGATCTACGGAACAATAAACTCTCTGGGAATATACCGCAGTTTGTAGATACCCAAGACATCAGTTTTCTTTTGTTGAGGGGGAACAGTTTAACGGGATATATTC  2800
NM_124306   : AATTCCGGACACATTTTTGGGAAGCATCCAGATACTTGATCTACGGAACAATAAACTCTCTGGGAATATACCGCAGTTTGTAGATACCCAAGACATCAGTTTTCTTTTGTTGAGGGGGAACAGTTTAACGGGATATATTC  1852
Cloned      : AATTCCGGACACATTTTTGGGAAGCATCCAGATACTTGATCTACGGAACAATAAACTCTCTGGGAATATACCGCAGTTTGTAGATACCCAAGACATCAGTTTTCTTTTGTTGAGGGGGAACAGTTTAACGGGATATATTC  1927

                 *     2820         *     2840         *     2860         *     2880         *     2900         *     2920         *     2940
Genomic-Seq : CAAGTACGCTTTGTGAATTCAGCAAGATGAGACTTTTAGATCTTTCTGATAACAAGCTCAATGGCTTCATACCTTCATGTTTCAATAATTTATCATTTGGACTGGCGAGAAAAGAGGAGATTACTAACTACTATGTTGCA  2940
NM_124306   : CAAGTACGCTTTGTGAATTCAGCAAGATGAGACTTTTAGATCTTTCTGATAACAAGCTCAATGGCTTCATACCTTCATGTTTCAATAATTTATCATTTGGACTGGCGAGAAAAGAGGAGATTACTAACTACTATGTTGCA  1992
Cloned      : CAAGTACGCTTTGTGAATTCAGCAAGATGAGACTTTTAGATCTTTCTGATAACAAGCTCAATGGCTTCATACCTTCATGTTTCAATAATTTATCATTTGGACTGGCGAGAAAAGAGGAGATTACTAACTACTATGTTGCA  2067

                 *     2960         *     2980         *     3000         *     3020         *     3040         *     3060         *     3080
Genomic-Seq : GTTGCATTGGAGAGCTTTTACTTGGGATTCTACAAATCCACATTTGTGGTTGAGAATTTCAGGTTAGACTACAGTAACTATTTTGAAATTGATGTAAAATTCGCAACCAAGCAAAGGTATGATTCTTATATAGGAGCATT  3080
NM_124306   : GTTGCATTGGAGAGCTTTTACTTGGGATTCTACAAATCCACATTTGTGGTTGAGAATTTCAGGTTAGACTACAGTAACTATTTTGAAATTGATGTAAAATTCGCAACCAAGCAAAGGTATGATTCTTATATAGGAGCATT  2132
Cloned      : GTTGCATTGGAGAGCTTTTACTTGGGATTCTACAAATCCACATTTGTGGTTGAGAATTTCAGGTTAGACTACAGTAACTATTTTGAAATTGATGTAAAATTCGCAACCAAGCAAAGGTATGATTCTTATATAGGAGCATT  2207

                 *     3100         *     3120         *     3140         *     3160         *     3180         *     3200         *     3220
Genomic-Seq : TCAGTTCAGCGAAGGAACACTTAATTCTATGTATGGCTTGGATCTGTCAAGCAATGAATTAAGTGGTGTTATCCCAGCAGAGCTTGGAGATCTCTTCAAACTAAGAGCCCTGAATTTGTCTCACAATTTCTTGTCGAGTC  3220
NM_124306   : TCAGTTCAGCGAAGGAACACTTAATTCTATGTATGGCTTGGATCTGTCAAGCAATGAATTAAGTGGTGTTATCCCAGCAGAGCTTGGAGATCTCTTCAAACTAAGAGCCCTGAATTTGTCTCACAATTTCTTGTCGAGTC  2272
Cloned      : TCAGTTCAGCGAAGGAACACTTAATTCTATGTATGGCTTGGATCTGTCAAGCAATGAATTAAGTGGTGTTATCCCAGCAGAGCTTGGAGATCTCTTCAAACTAAGAGCCCTGAATTTGTCTCACAATTTCTTGTCGAGTC  2347

                 *     3240         *     3260         *     3280         *     3300         *     3320         *     3340         *     3360
Genomic-Seq : ATATACCGGATAGCTTCTCTAAATTGCAGGACATTGAGAGCCTTGATCTTTCTTATAACATGTTACAAGGAAGCATTCCTCACCAGTTAACCAACCTCACTTCTCTTGCTATCTTCAACGTCTCTTACAACAATTTATCA  3360
NM_124306   : ATATACCGGATAGCTTCTCTAAATTGCAGGACATTGAGAGCCTTGATCTTTCTTATAACATGTTACAAGGAAGCATTCCTCACCAGTTAACCAACCTCACTTCTCTTGCTATCTTCAACGTCTCTTACAACAATTTATCA  2412
Cloned      : ATATACCGGATAGCTTCTCTAAATTGCAGGACATTGAGAGCCTTGATCTTTCTTATAACATGTTACAAGGAAGCATTCCTCACCAGTTAACCAACCTCACTTCTCTTGCTATCTTCAACGTCTCTTACAACAATTTATCA  2487

                 *     3380         *     3400         *     3420         *     3440         *     3460         *     3480         *     3500
Genomic-Seq : GGCATCATTCCCCAAGGAAAGCAATTTAACACCTTCGACGAGAACAGCTACTTAGGAAATCCTCTTCTCTGTGGACCGCCAACCGATACAAGCTGTGAAACTAAGAAGAACTCAGAGGAAAATGCCAATGGAGGAGAAGA  3500
NM_124306   : GGCATCATTCCCCAAGGAAAGCAATTTAACACCTTCGACGAGAACAGCTACTTAGGAAATCCTCTTCTCTGTGGACCGCCAACCGATACAAGCTGTGAAACTAAGAAGAACTCAGAGGAAAATGCCAATGGAGGAGAAGA  2552
Cloned      : GGCATCATTCCCCAAGGAAAGCAATTTAACACCTTCGACGAGAACAGCTACTTAGGAAATCCTCTTCTCTGTGGACCGCCAACCGATACAAGCTGTGAAACTAAGAAGAACTCAGAGGAAAATGCCAATGGAGGAGAAGA  2627

                 *     3520         *     3540         *     3560         *     3580         *     3600         *     3620         *     3640
Genomic-Seq : AGATGACAAAGAAGTTGCTATTGACATGTTGGTCTTCTATTGGAGTACTGCTGGAACTTATGTGACTGCATTGATAGGTATTCTTGTACTTATGTGTGTTGATTGTTCTTGGCGTCGAGCATGGCTCCGCCTTGTCGATG  3640
NM_124306   : AGATGACAAAGAAGTTGCTATTGACATGTTGGTCTTCTATTGGAGTACTGCTGGAACTTATGTGACTGCATTGATAGGTATTCTTGTACTTATGTGTGTTGATTGTTCTTGGCGTCGAGCATGGCTCCGCCTTGTCGATG  2692
Cloned      : AGATGACAAAGAAGTTGCTATTGACATGTTGGTCTTCTATTGGAGTACTGCTGGAACTTATGTGACTGCATTGATAGGTATTCTTGTACTTATGTGTGTTGATTGTTCTTGGCGTCGAGCATGGCTCCGCCTTGTCGATG  2767

                 *     3660         *
Genomic-Seq : CTTTCATTGCCTCTGCGAAAAGTAAGTTGGCTTAA  3675
NM_124306   : CTTTCATTGCCTCTGCGAAAAGTAAGTTGGCTTAA  2727
Cloned      : CTTTCATTGCCTCTGCGAAAAGTAAGTTGGCTTAA  2802
```

Figure S5. Osmotic effects on the seeds germination of WT and *AtRLP28-OX* lines using mannitol. Germination percentages of WT and three independent *AtRLP28-OX* seeds grown for 2d on the 1/2 MS medium supplemented with 200mM and 300mM mannitol.

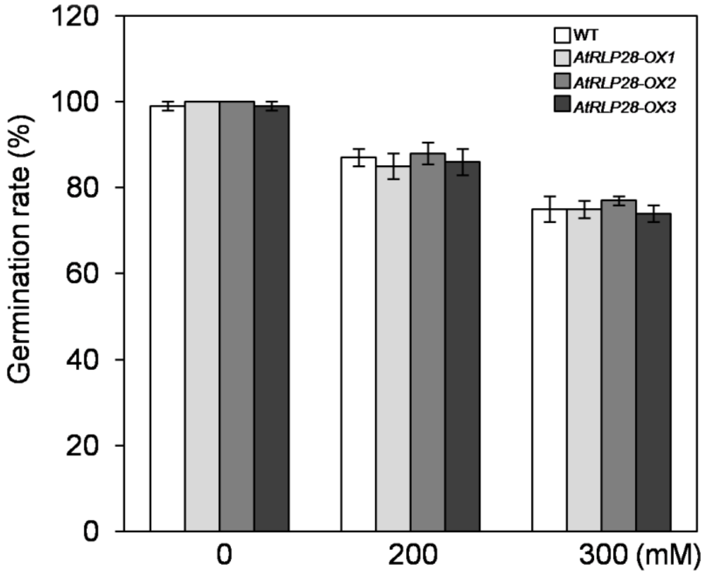

Table S1. A list of quantitative real-time PCR primers used in this study.

Gene name	Forward primer	Reverse primer
AtRLP23	ATGGAGTCTGGTGCGATAACT	GAAGAGGCTACTGTTGGACTT
AtRLP28	AGTCTGGTGCGATAACTCGAC	CAGAATAGGCTACTGTTGGGA
AtRLP30	ACTGCACACAAACACGAG	TCAACGAGCACTTGTGGTG
AtRLP33	TGTCCAAGTCCTCTGAAGACA	CTGCACATAAGGTCTATCTCG
AtRLP37	ACACTTTCGCTTCTCCCACAC	TGGCTCCCGTTGGAATGGAT
RD29A	GACGGGATTTGACGGAGAAC	CCGCCACATAATCTCTACCC
RAB18	CGATCCAGCAGCAGTATGAC	TTCGAAGCTTAACGGCCACC
Actin2	GACCAGCTCTTCCATCGAGAA	CAAACGAGGGCTGGAACAAG

Table S2. *AtRLP* genes which locate close to an *RLK* gene.

AtRLP gene (AGI code/*AtRLP*)	*RLKs*	
	AGI code	Subfamily
At1g17240 (*AtRLP2*)<br>At1g17250 (*AtRLP3*)	At1g17230	LRR XI
At1g28340 (*AtRLP4*)	At1g28390	CR4L
	At1g28440	LRR XI
At1g34290 (*AtRLP5*)	At1g34210	LRR II
	At1g34300	SD-2
At2g33080 (*AtRLP28*)	At2g33170	LRR XI
At3g24900 (*AtRLP39*)	At3g24790	RLCK VII
At3g49750 (*AtRLP44*)	At3g49670	LRR XI
At5g25910 (*AtRLP52*)	At5g25930	LRR XI
At5g45770 (*AtRLP55*)	At5g45780	LRR II
	At5g45800	LRR VII
	At5g45840	LRR VI

Table S3. *AtRLP* genes displaying no transcriptional responses to the experimental conditions.

AtRLP **genes displaying no response to abiotic stress**	***AtRLP*** **genes displaying no response to biotic stress**	***AtRLP*** **genes displaying no response to hormonal treatments**	***AtRLP*** **genes displaying no response to any condition**
AtRLP5	*AtRLP4*	*AtRLP2*	*AtRLP8*
AtRLP8	*AtRLP8*	*AtRLP4*	
AtRLP11	*AtRLP14*	*AtRLP5*	
AtRLP15	*AtRLP16*	*AtRLP6*	
AtRLP25	*AtRLP17*	*AtRLP8*	
AtRLP43	*AtRLP29*	*AtRLP9*	
AtRLP48	*AtRLP44*	*AtRLP11*	
AtRLP55		*AtRLP13*	
AtRLP56		*AtRLP15*	
		AtRLP16	
		AtRLP18	
		AtRLP20	
		AtRLP24	
		AtRLP28	
		AtRLP29	
		AtRLP31	
		AtRLP35	
		AtRLP36	
		AtRLP40	
		AtRLP43	
		AtRLP44	
		AtRLP45	
		AtRLP47	
		AtRLP48	
		AtRLP52	
		AtRLP55	
		AtRLP56	

Table S9. Overview of the cloning results of *AtRLP* genes.

**Cloned *AtRLP* genes**		**Un-cloned *AtRLP* genes (4)**	**Pseudogenes (2)**
**Cloned *AtRLP* genes identical to the predictions in TAIR (44)**	**Cloned *AtRLP* genes different from the predictions in TAIR (7)**		
AtRLP2; *AtRLP3*; *AtRLP5* *AtRLP6*; *AtRLP7*; *AtRLP9* *AtRLP10*; *AtRLP11*; *AtRLP12* *AtRLP14*; *AtRLP16*; *AtRLP17* *AtRLP19*; *AtRLP22*; *AtRLP23* *AtRLP25*; *AtRLP26*; *AtRLP27* *AtRLP28*; *AtRLP29*; *AtRLP30* *AtRLP31*; *AtRLP32*; *AtRLP33* *AtRLP34*; *AtRLP35*; *AtRLP36* *AtRLP37*; *AtRLP38*; *AtRLP39* *AtRLP41*; *AtRLP42*; *AtRLP43* *AtRLP44*; *AtRLP45*; *AtRLP46* *AtRLP47*; *AtRLP48*; *AtRLP50* *AtRLP51*; *AtRLP53*; *AtRLP54* *AtRLP55*; *AtRLP57*	*AtRLP4*; *AtRLP13*; *AtRLP20* *AtRLP24*; *AtRLP40*; *AtRLP52* *AtRLP56*	*AtRLP1*; *AtRLP15*; *AtRLP21* *AtRLP8*	*AtRLP18*; *AtRLP49*

Table S10. Summary of the *AtRLP*-OX transgenic plants.

Gene name	Destination vectors	Genotypes that been transformed	Number of homozygous *AtRLP*-OX lines
AtRLP2	pGD625	Col-0	3
		L*er*	3
AtRLP3	pGD625	Col-0	3
		L*er*	3
		rlp10-1	
		clv2-1	
AtRLP4	pGD625	Col-0	4
AtRLP5	pGD625	Col-0	3
AtRLP6	pGD625	Col-0	3
AtRLP7	pGD625	Col-0	3
AtRLP9	pGD625	Col-0	3
AtRLP11	pGD625	Col-0	3
		L*er*	3
		rlp10-1	
		clv2-1	
AtRLP12	pGD625	Col-0	3
		L*er*	3
AtRLP13	pGD625	Col-0	3
AtRLP14	pGD625	Col-0	3
AtRLP16	pGD625	Col-0	3
AtRLP17	pGD625	Col-0	3
AtRLP19	pGD625	Col-0	3
AtRLP20	pGD625	Col-0	3
AtRLP22	pGD625	Col-0	3
AtRLP23	pGD625	Col-0	3
AtRLP24	pGD625	Col-0	3
AtRLP25	pGD625	Col-0	3
AtRLP26	pGD625	Col-0	3
AtRLP27	pGD625	Col-0	3
AtRLP28	pGD625	Col-0	4
AtRLP29	pGD625	Col-0	4
AtRLP30	pGD625	Col-0	3
AtRLP31	pGD625	Col-0	3
AtRLP32	pFAST-R02	Col-0	3
AtRLP33	pGD625	Col-0	5
AtRLP34	pGD625	Col-0	3
AtRLP35	pGD625	Col-0	3
AtRLP36	pGD625	Col-0	3

	AtRLP37	pGD625	Col-0	3
	AtRLP38	pGD625	Col-0	3
	AtRLP39	pGD625	Col-0	3
	AtRLP40	pGD625	Col-0	3
	AtRLP41	pGD625	Col-0	3
	AtRLP42	pGD625	Col-0	3
	AtRLP43	pGD625	Col-0	3
	AtRLP44	pGD625	Col-0	3
	AtRLP45	pGD625	Col-0	3
	AtRLP46	pB2GW7, 0	Col-0	3
	AtRLP47	pGD625	Col-0	3
	AtRLP48	pGD625	Col-0	3
	AtRLP50	pGD625	Col-0	3
	AtRLP51	pGD625	Col-0	3
	AtRLP52	pGD625	Col-0	3
	AtRLP53	pGD625	Col-0	3
	AtRLP54	pGD625	Col-0	3
	AtRLP55	pGD625	Col-0	3
	AtRLP56	pGD625	Col-0	3
	AtRLP57	pGD625	Col-0	3
**Total**	**51**			**167**
